# Nisin bacteriocin blocks *T. denticola*-triggered MMP2 activation and pathogen internalization via TLR2

**DOI:** 10.1038/s41598-026-43673-8

**Published:** 2026-03-11

**Authors:** Pachiyappan Kamarajan, Allan Radaic, Hossein Beheshti, Mishri Parikh, Sarah Lo, J Christopher Fenno, Yvonne L. Hernandez Kapila

**Affiliations:** 1 Department of Biosystems and Function, School of Dentistry, University of California Los Angeles, Los Angeles, CA USA; 2 Department of Biologic and Materials Sciences & Prosthodontics, School of Dentistry, University of Michigan, Ann Arbor, MI USA

**Keywords:** Periodontal ligament cells, Treponema denticola, Nisin, Antimicrobial peptide, Toll-like receptor-2, Matrix metalloproteinases 2 protein, Dentilisin/PrtP, PrtP, Diseases, Microbiology

## Abstract

We examined *Treponema denticola*-induced dysregulation of toll-like receptor (TLR) signaling and its downstream effects on matrix metalloproteinase 2 (MMP2) expression and activation in periodontal cells, and whether the antimicrobial peptide nisin could modulate this process. *T. denticola* and its outer membrane protease dentilisin/PrtP activated MMP2, whereas a dentilisin/PrtP-deficient *T. denticola* mutant did not, and nisin attenuated this response. Dentilisin/PrtP directly activated recombinant MMP2 in vitro. *T. denticola*-mediated MMP2 activation was TLR2-dependent, since TLR2 suppression abrogated MMP2 activation, reduced MMP2 mRNA expression, and reduced *T. denticola* internalization. Nisin significantly inhibited *T. denticola* internalization. Computational modeling suggests dentilisin/PrtP directly binds pro-MMP2, whereas nisin binds dentilisin/PrtP but not pro-MMP2. Thus, nisin may interfere with MMP2 activation by binding to dentilisin/PrtP. These findings reveal a novel mechanism by which *T. denticola* and nisin regulate MMP2, and underscore nisin’s potential as a therapeutic for modulating bacterial-mediated tissue damage during periodontal disease.

**Supplementary Information:**

The online version contains supplementary material available at 10.1038/s41598-026-43673-8.

## Introduction

Periodontal disease constitutes a significant public health burden, affecting approximately 30% of the adult population in the United States^1,2^ and similar proportions globally^3^. This disease is characterized by microbial dysbiosis, which subsequently triggers chronic host inflammatory responses and cellular catabolic processes. These processes culminate in the apical migration of the junctional epithelium, chronic periodontal tissue destruction mediated by matrix metalloproteinases (MMPs), alveolar bone loss, and ultimately, tooth loss^4–8^. While gingival inflammation precedes periodontitis, the molecular mechanisms that drive attachment loss, periodontal ligament degradation, and alveolar bone loss are more complex and distinct from those of gingival inflammation alone^9,10^. The specific cellular responses, including those resulting from microbe-host cell interactions, that facilitate the transition from reversible gingival inflammation to irreversible periodontal tissue destruction are of particular interest and reside within the periodontal ligament (PDL).

Matrix metalloproteinases (MMPs) play a key role in periodontal tissue destruction by mediating extracellular matrix degradation, basement membrane disruption, and connective tissue remodeling. Recent reviews highlight that dysregulated MMP activity is a major factor driving periodontal disease progression, linking chronic inflammation to irreversible tissue breakdown^11^. Among the MMPs involved in periodontitis, matrix metalloproteinase-2 (MMP2) is especially important because of its ability to degrade type IV collagen and other basement membrane components, which is a critical step in epithelial barrier disruption and connective tissue invasion^11,12^. In addition to matrix degradation, MMP2 contributes to angiogenesis, cell migration, and tissue remodeling—processes essential to both periodontal disease progression and healing^13^. Experimental studies have shown that periodontal pathogens upregulate and activate MMP-2 in periodontal ligament cells, implicating MMP-2 as a key mediator of pathogen-induced tissue destruction^14,15^. Clinically, increased MMP2 activity has been linked to periodontal tissue destruction and disease severity, supporting its role as a key effector protease distinct from neutrophil-derived collagenases^16^. Overall, these features establish MMP2 as a central mediator of basement membrane disruption and tissue remodeling in periodontitis, justifying its focused study compared to other MMP family members.

Spirochetes are particularly associated with severe periodontitis^17^, and they preferentially localize in the deepest regions of the lesions^18^. *T. denticola* is a key oral pathobiont^19^ whose abundance increases with periodontal inflammation and contributes to periodontal dysbiosis^17,20,21^. It also serves as an established model for studying spirochete biology and *Treponema*-host interactions in the context of periodontal disease. Although periodontal pathogens, including *T. denticola*, are regarded as primary etiological factors, the literature indicates that direct causation of tissue destruction arises from host responses to a dysbiotic bacterial community^22–26^. Clinical data regarding the increased presence of *T*. *denticola* in periodontal lesions, together with basic and in vivo studies involving the role of *T*. *denticola* and its virulence factors, suggest that it plays a pivotal role in driving periodontal disease progression^14,25,27–32^.

*T. denticola* contributes to periodontal pathogenesis through multiple virulence mechanisms, including adherence to host tissues via TLR2^33^ and coaggregation with other oral microbes^34,35^. Its helical shape and periplasmic flagella confer exceptional motility, allowing penetration of biofilms, invasion of periodontal tissues, and close interaction with host cells to deliver virulence factors^35,36^. Motility and chemotaxis also guide *T. denticola* toward inflammatory mediators and tissue breakdown products, promoting persistence and pathogenic synergy within polymicrobial biofilms^36,37^. Notably, *T. denticola’s* two outer membrane protein complexes^38–40^ are implicated in pathogenic interactions with host cells; namely a protease complex comprising three lipoproteins (dentilisin/PrtP) and the trimeric major surface protein (Msp)^41–43^. Among the various effector molecules produced by *T. denticola*, dentilisin/PrtP stands out as a significant virulence factor. It induces several cytopathic effects consistent with the pathophysiology of periodontal disease^35,40,44,45^. Notable examples of its effects include facilitating adhesion, degrading endogenous extracellular matrix (ECM) substrates^35^, penetrating tissues^46^, evading complement^47^, ectopically activating host MMPs^15,32^, and breaking down host chemokines and cytokines, such as interleukin-1β (IL-1β) and interleukin-6 (IL-6), primarily due to its strong proteolytic activity^48^. Therefore, while establishing *T. denticola* as a direct instigator of gingival inflammation may be challenging, evidence supports a model wherein *T. denticola* proliferation, propelled by a changing nutrient environment resulting from an inflammogenic, dysbiotic microbial community, disrupts normal tissue responses, thereby facilitating subsequent tissue destruction^22,49^. Consequently, elucidating the mechanisms by which *T. denticola* promotes the disruption of tissue homeostasis will enhance our understanding of periodontal disease pathogenesis and identify potentially novel therapeutic entry points.

Nisin, a naturally occurring bacteriocin produced by *Lactococcus lactis* and commonly used food preservative, has been recognized by the Food and Drug Administration (FDA) as Generally Recognized As Safe (GRAS)^50^ and has garnered interest beyond food preservation due to its broad-spectrum antimicrobial properties. Several studies, including our own, have shown that nisin maintains a robust safety profile in both humans and animals while demonstrating significant effectiveness against various non-foodborne pathogens, including oral pathogens^51–57^. Nisin also disrupts the structure and function of pathogenic oral biofilms^51,58,59^, a critical step toward restoring oral microbial balance. These biofilms contribute to oral disease by shielding bacteria from environmental stresses, facilitating horizontal gene transfer, and altering the structure of the commensal microbial community^60,61^. Additionally, nisin can reduce oral and systemic inflammation^54–56,62–69^, and it is being tested in a human clinical trial for oral squamous cell carcinoma (OSCC) patients (ClinicalTrials.gov; NCT06097468, Registration date: 10-18-23).

Given our previous finding that *T. denticola* dentilisin/PrtP-triggered-Toll-like receptor-2 (TLR2) activation upregulated a tissue destructive pathways of transcriptional activation of MMPs^32^, and since nisin has antimicrobial effects against *T. denticola*^51,57^, this study focused on examining nisin’s ability to modulate *T. denticola’s* activation of MMP2 expression.

## Results

### Nisin inhibits T. denticola-induced-MMP2 activation

To characterize *T. denticola*-host interactions in PDL cells, we examined *T. denticola’s* impact on MMP2 protease activity and the degree to which nisin modulates this activity. The concentration of nisin used in this study was selected based on prior publications demonstrating its biological activity while avoiding nonspecific cytotoxicity. Specifically, nisin concentrations were selected according to our previous work^51,59,70^. These studies showed that nisin at low microgram (µg/ml) levels is non-toxic to normal oral cells while effectively modulating inflammatory signaling, inhibiting bacterial pathogenicity, and reducing disease-related cellular responses. Notably, these concentrations produced biological effects without harming cell viability, supporting their suitability for mechanistic studies.

**Fig. 1:**
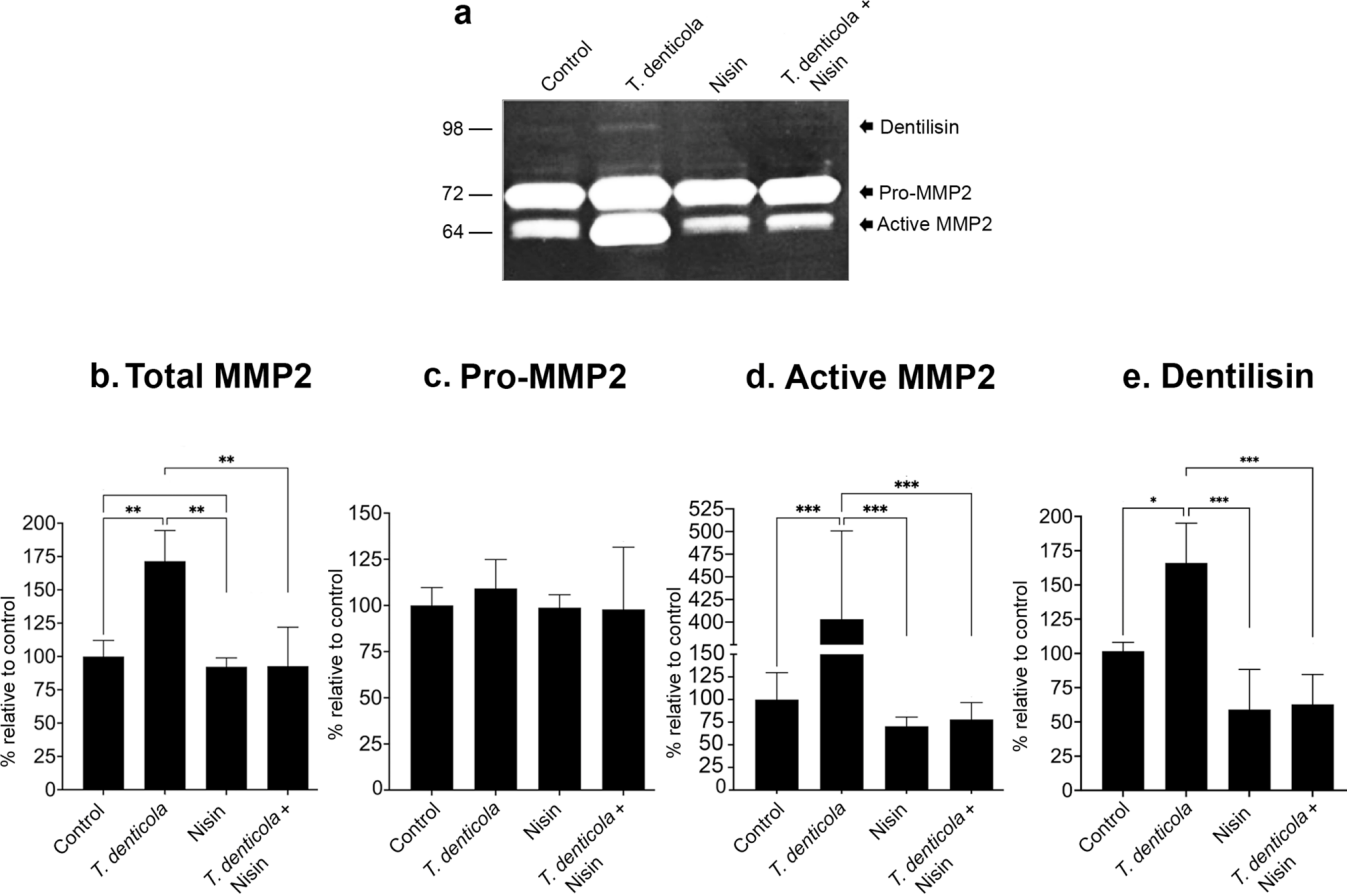
Nisin inhibits *T. denticola*-induced-MMP2 activation in PDL cells. (**a**) Representative gelatin zymogram showing MMP2 levels in PDL cells exposed to wild-type *T. denticola* (35405) (50 MOI) for 2 h, washed three times with PBS, followed by treatment with nisin (100 µg/mL) for 24 h. Conditioned media were analyzed by gelatin zymography. Quantitative analysis of band intensities for total MMP2 (**b**), Pro-MMP2 (c), active MMP2 (d) and dentilisin/PrtP (e). Data are representative of at least three independent experiments. Intergroup differences were analyzed by the analysis of variance (ANOVA) and Tukey’s post hoc test. *p < 0.05, ** *p* < 0.01, *** *p* < 0.001, **** *p* < 0.0001. The uncropped gel image is presented in Supplementary Information as Fig. 1a.

PDL cells were exposed to wild-type *T. denticola* (35405) (50 MOI) for 2 h, washed 3 times with PBS, and then treated with nisin (100 µg/mL) for 24 h. Conditioned media were collected and analyzed for secreted MMP2 levels using Gelatin zymography. As previously demonstrated by us and others^15,35,71–73^, PDL cells expressed basal levels of pro-MMP2 and active MMP2. Challenge with dentilisin/PrtP-secreting *T. denticola* significantly enhanced total MMP2 (Fig. 1a and b) and active MMP2 (Fig. 1a and d) in PDL cells, whereas nisin significantly attenuated this activation. Nisin alone did not impact active MMP2 levels compared to control conditions. Pro-MMP2 levels remained statistically unchanged across all treatments when compared to baseline. (Fig. 1a and c). These findings indicate that *T. denticola* secreting dentilisin/PrtP mediates MMP2 activation in PDL cells, a process that can be effectively inhibited by nisin. In addition, we noted that dentilisin/PrtP exhibits gelatinolytic activity, which is attenuated by nisin treatment (Fig. 1a and e).

### Purified* T. denticola* dentilisin/PrtP directly activates recombinant MMP2 in vitro

**Fig. 2:**
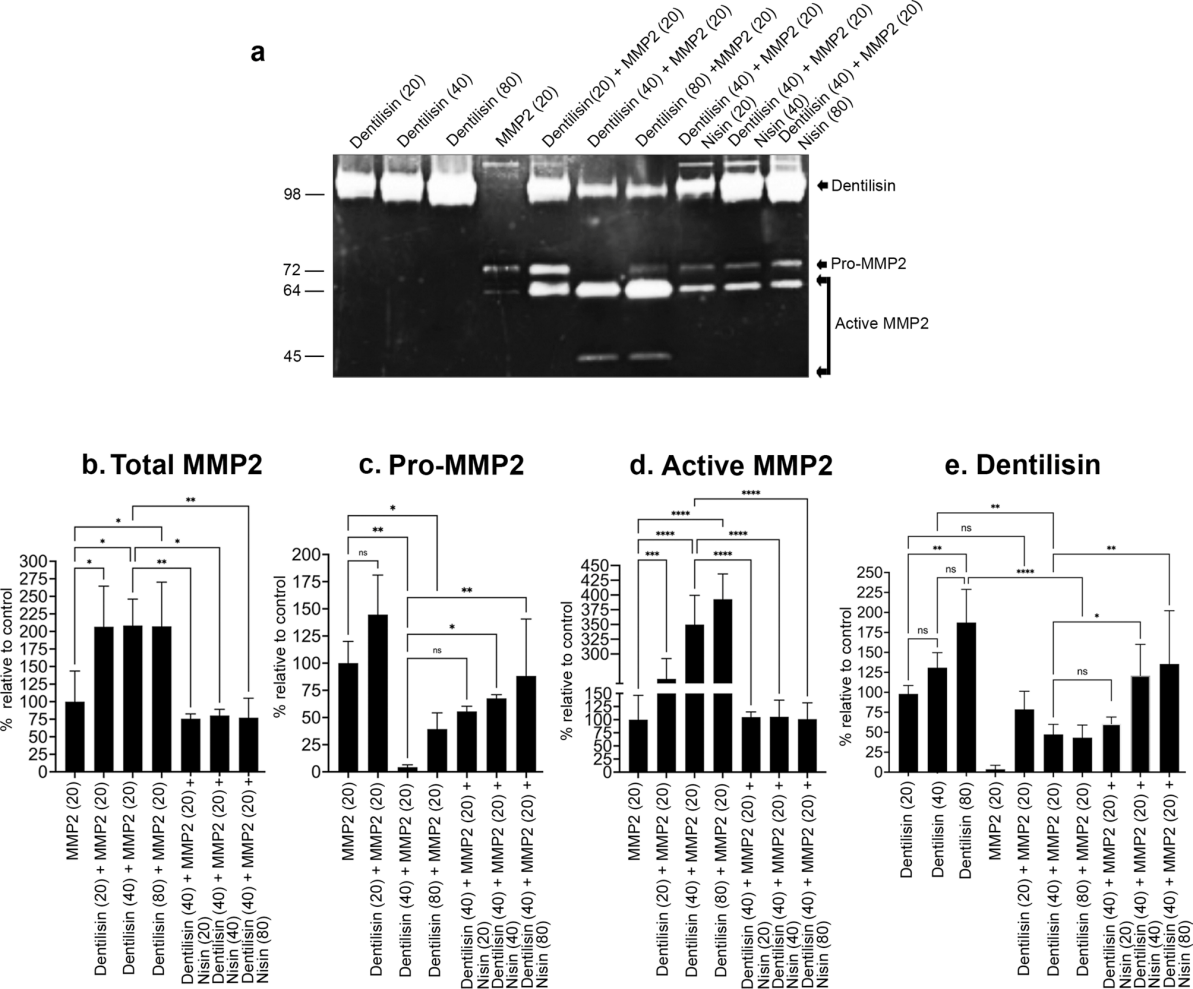
*T. denticola* purified dentilisin/PrtP directly activates recombinant MMP2 in vitro, and nisin attenuates this activation. (**a**) Representative gelatin zymogram of recombinant Pro-MMP2 (20 ng) incubated with increasing concentrations of purified dentilisin/PrtP (20, 40, or 80 ng) in the presence or absence of nisin (20, 40, or 80 µg). Quantitative analysis of band intensities for total MMP2 (**b**), Pro-MMP2 (**c**), active MMP2 (**d**) and dentilisin/PrtP (**e**). Data are representative of at least three independent experiments. Intergroup differences were analyzed by the analysis of variance (ANOVA) and Tukey’s post hoc test. *p < 0.05, ** *p* < 0.01, *** *p* < 0.001, **** *p* < 0.0001. The uncropped gel image is presented in Supplementary Information as Fig. 2a.

**Fig. 3:**
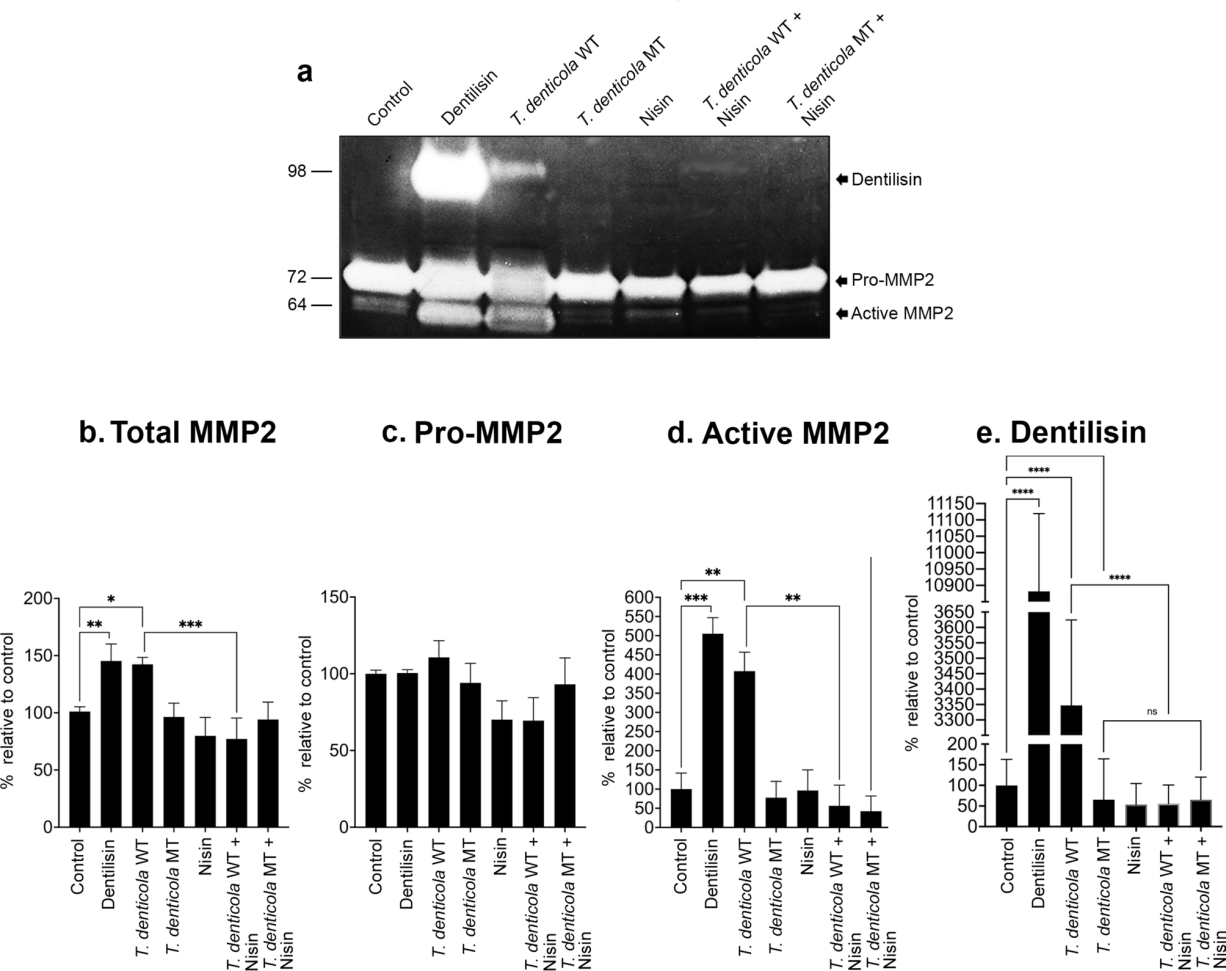
Dentilisin/PrtP-deficient *T. denticola* does not activate MMP2 and is unaffected by nisin in PDL cells. (**a**) Representative gelatin zymogram showing MMP2 levels in PDL cells exposed to purified dentilisin/PrtP (50 ng/ml), wild-type *T. denticola* (WT) (35405) (50 MOI) or mutant *T. denticola* (MT) (50 MOI) for 2 h and treated with nisin (100 µg/ml) for 24 h. Conditioned media were analyzed by gelatin zymography. Quantitative analysis of band intensities for total MMP2 (**b**), Pro-MMP2 (**c**), active MMP2 (**d**) and dentilisin/PrtP (**e**). Data are representative of at least three independent experiments. Intergroup differences were analyzed by the analysis of variance (ANOVA) and Tukey’s post hoc test. *p < 0.05, ** *p* < 0.01, *** *p* < 0.001, **** *p* < 0.0001. The uncropped gel image is presented in Supplementary Information as Fig. 3a.

Since dentilisin/PrtP, a key pathogenic effector molecule of *T. denticola*, can significantly increase MMP2 activation in PDL cells^15,35^, and since dentilisin/PrtP is a serine protease, and serine proteases are known to activate MMPs, we hypothesized that dentilisin/PrtP might directly activate MMP2. To test this hypothesis, we incubated recombinant MMP2 protein with increasing concentrations of purified dentilisin/PrtP, with or without nisin, in vitro, and then analyzed potential MMP2 activation using zymography. The dentilisin concentration was determined based on foundational studies by Fenno and colleagues, which identified dentilisin as a potent *T. denticola* virulence factor that activates host receptors and downstream inflammatory and proteolytic pathways at low concentrations^15,35,40^ Previous research demonstrated that dentilisin at these levels is sufficient to trigger TLR2-dependent signaling, cytokine production, matrix metalloproteinase activation, and host cell responses, without causing nonspecific proteolysis or cytotoxicity^32^. Therefore, the concentrations used in this study align with previously published functional ranges, allowing direct comparison with earlier work and ensuring that the observed effects are due to the specific biological activity of nisin and dentilisin rather than off-target or toxic effects. Overall, the concentration of dentilisin was selected to fall within validated, biologically active ranges, ensuring that the observed effects result from specific modulation of dentilisin-mediated pathogenic mechanisms rather than from off-target or toxic effects. Our results demonstrated that dentilisin/PrtP directly elicited a dose-dependent activation of MMP2, as evidenced by increased levels of the active MMP2 accompanied by a corresponding decrease in pro-MMP2. Notably, treatment with nisin markedly attenuated this dentilisin/PrtP-induced activation of MMP2 (Fig. 2a, c and d). Dentilisin/PrtP also significantly increased total MMP2, an activity effectively counteracted by nisin. (Fig. 2a and b). These findings demonstrate that dentilisin/PrtP directly mediates MMP2 activation, which can be mitigated by nisin treatment. Furthermore, nisin enhanced dentilisin/PrtP’s gelatin-degrading activity dose-dependently in the absence of host cells *in* vitro (Fig. 2e) but inhibited it in the presence of cells (Figs. 1 and 3), suggesting that PDL cell–derived factors modulate nisin’s effect on dentilisin/PrtP activity and mediate altered protein-protein interactions.

### Purified MMP2 attenuates dentilisin/PrtP-gelatin degrading activity in vitro and nisin restores it

Purified MMP2 when incubated with increasing concentrations of purified dentilisin/PrtP, unexpectedly attenuated dentilisin/PrtP’s gelatin-degrading activity dose-dependently (Fig. 2a and e), whereas in the presence of PDL cells and recombinant MMP2, dentilisin/PrtP activity was maintained (Supplementary Fig. 1). These findings suggest that PDL cell–derived factors modulate MMP2 activity, thereby limiting dentilisin/PrtP degradation.

### Dentilisin/PrtP-deficient *T. denticola* does not activate MMP2 and is unaffected by nisin in this context

To confirm the importance of dentilisin/PrtP in MMP2 activation in vivo in cells and in the context of nisin, we investigated the impact of treatment with a *T. denticola* dentilisin/PrtP mutant compared to the counterpart wildtype *T. denticola* strain in the presence and absence of nisin treatment. PDL cells were exposed to purified dentilisin/PrtP (50 ng/ml), wild-type *T. denticola* (WT) (35405) (50 MOI) or mutant *T. denticola* (MT) (50 MOI) for 2 h and treated with nisin (100 µg/ml) for 24 h. Conditioned media were analyzed by gelatin zymography. Dentilisin/PrtP-expressing *T. denticola* promoted the activation of MMP2 in PDL cells, whereas the dentilisin/PrtP-deficient mutant did not (Fig. 3a and d). As previously noted in Fig. 1, nisin blocked the MMP2 activation triggered by *T. denticola*, but it had no impact on MMP2 activation in the context of the dentilisin/PrtP-deficient mutant (Fig. 3a and d). Treatment with dentilisin/PrtP or dentilisin/PrtP-expressing *T. denticola* also enhanced active and total MMP2 (Fig. 3a and b), and Pro-MMP2 levels remained statistically unchanged across all treatments compared to baseline in PDL cells (Fig. 3a and c). Nisin alone did not significantly affect total-MMP2, pro-MMP2 or active MMP2 compared to the control. Furthermore, nisin inhibited the gelatinolytic activity of dentilisin/PrtP (Fig. 3e).

### *T. denticola*-triggered activation of MMP2 is mediated via TLR2

**Fig. 4:**
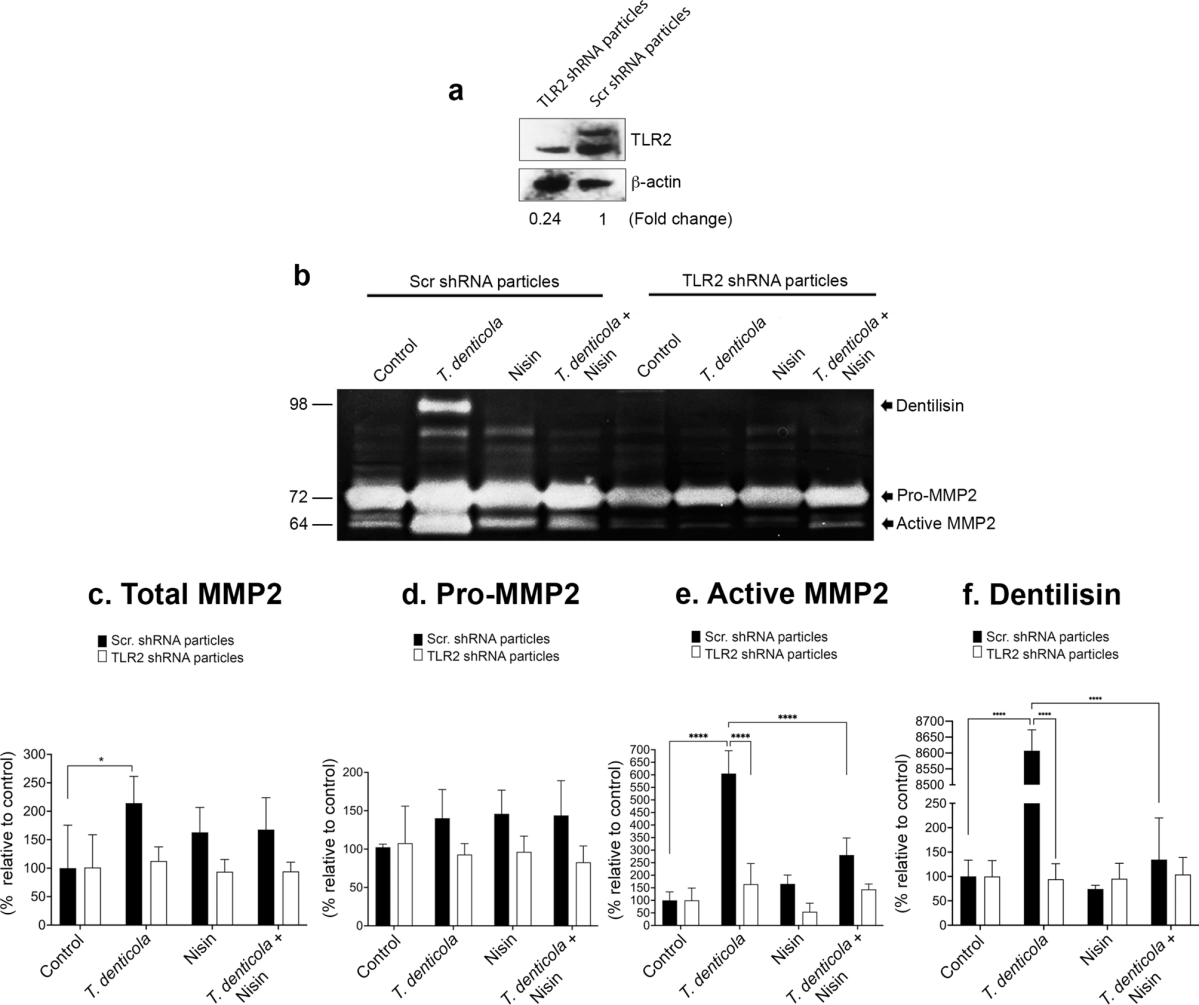
*T. denticola*-triggered activation of MMP2 and nisin effects are mediated via TLR2 in PDL cells. PDL cells were transduced with TLR2 shRNA or scramble shRNA lentiviral particles in serum-free media, then selected in puromycin. (**a**) Immunoblot showing TLR2 protein levels in cells transduced with scramble shRNA or TLR2 shRNA lentiviral particles. (**b**) Representative gelatin zymogram showing MMP2 levels in PDL cells exposed to wild-type *T. denticola* (35405) (50 MOI) for 2 h, washed three times with PBS, treated with gentamicin (50 µg/ml) for 1 h, washed three times again with PBS, and then treated with nisin (100 µg/ml) for 24 h. Conditioned media were analyzed by gelatin zymography. Quantitative analysis of band intensities for total MMP2 (**c**), Pro-MMP2 (**d**), active MMP2 (**e**) and dentilisin/PrtP (**f**). Data are representative of at least three independent experiments. Intergroup differences were analyzed by the analysis of variance (ANOVA) and Tukey’s post hoc test. *p < 0.05, ** *p* < 0.01, *** *p* < 0.001, **** *p* < 0.0001. The uncropped blot and gel images are presented in Supplementary Information as Figs. 4a and b.

Since *T. denticola* interacts with Toll-like receptors, including TLR2, to mediate its cytopathic effects, we investigated the extent to which *T. denticola* activates MMP2 via TLR2. To this end, we stably suppressed the expression of TLR2 in PDL cells and then examined how this impacted *T. denticola’s* ability to activate MMP2 expression. Using a TLR2 shRNA approach, we suppressed TLR2 expression ~ 76% in PDL cells compared to the shRNA control (Fig. 4a). Cells transduced with scrambled shRNA or TLR2 shRNA were exposed to wild-type *T. denticola* (35405) (50 MOI) for 2 h, washed three times with PBS, treated with gentamicin (50 µg/ml) for 1 h, washed three times again with PBS, and then treated with nisin (100 µg/ml) for 24 h. Conditioned media were analyzed by gelatin zymography. In the context of TLR2 suppression, *T. denticola*-mediated activation of MMP2 was suppressed by 50% compared to the shRNA control, demonstrating that *T. denticola* promotes MMP2 activation in PDL cells through a TLR2-dependent process (Fig. 4b and e). Since TLR2 suppression abrogated *T. denticola*-activation of MMP2, nisin’s effect on this process could not be discerned. In the context of TLR2 suppression, Total MMP2 and Pro-MMP2 levels did not change significantly across treatments relative to baseline (Fig. 4b-d). Furthermore, TLR2 suppression unexpectedly suppressed dentilisin/PrtP’s gelatin-degradating activity but not in control conditions (Fig. 4b and e). This unexpected finding was further and subsequently explored in the context of cellular internalization experiments.

### Suppression of TLR2 inhibits the internalization of *T. denticola* and nisin into the cytosol, and nisin attenuates *T. denticola* internalization

**Fig. 5:**
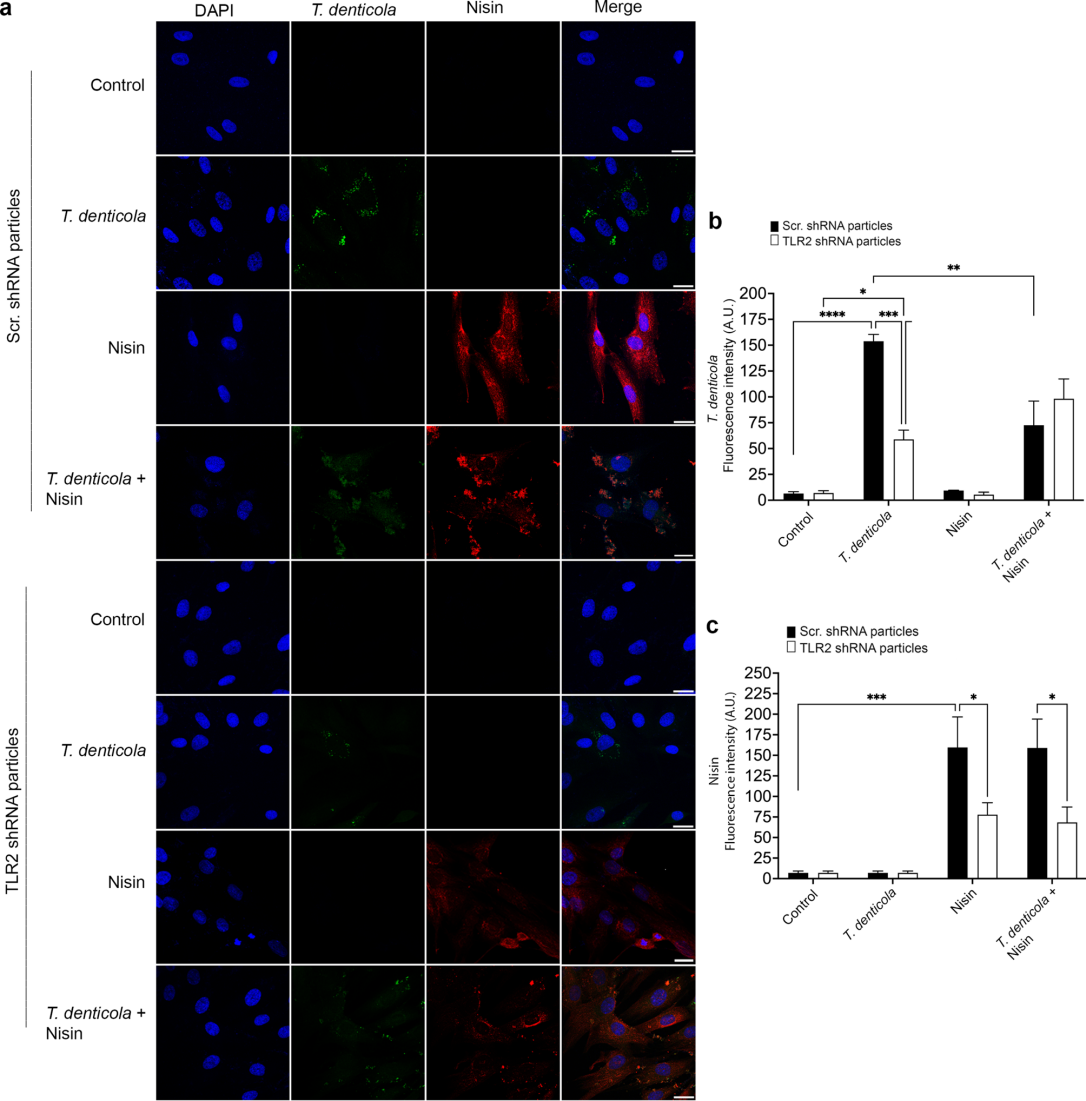
Suppression of TLR2 inhibits the internalization of *T. denticola* and nisin into the nucleus in PDL cells. PDL cells were exposed to Syto 9-stained *T. denticola* (35405) (50 MOI) for 4 h, washed three times with PBS, treated with gentamicin (50 µg/ml) for 1 h, washed three times again with PBS, and then treated with nisin for (100 µg/ml) 24 h. (**a**) Confocal microscopy images show co-localization of T. *denticola* (green) and nisin (Red) inside the cell; suppression of TLR2 reduced their internalization (Scale bar = 20 μm). Quantitative analysis of fluorescence intensity for *T. denticola* (**b**) and nisin (**c**).

Upon establishing that TLR2, a cell surface receptor that mediates intracellular signaling, plays a pivotal role in the regulation of *T. denticola*-induced MMP activation, we sought to further investigate the degree to which TLR-mediated cellular internalization processes might be mediating these effects. Therefore, in this context, cells were first exposed to SYTO 9-stained *T. denticola* for 4 h, washed three times with PBS, then treated with gentamicin (50 µg/ml) for 1 h to inactivate any remaining extracellular bacteria, then washed again, and treated with nisin (100 µg/ml) for 24 h. Using confocal microscopy, we observed that *T. denticola* and nisin were internalized into PDL cells, showing a dispersed distribution of bacteria and nisin in the cytosol (Fig. 5a). Suppression of TLR2 hindered internalization of both nisin and *T. denticola* into this region, indicating that their internalization is TLR2-dependent (Fig. 5a). Quantification of the fluorescence intensity further confirmed the TLR2-dependent internalization of *T. denticola* and nisin, as shown in Fig. 5b and c. Furthermore and importantly, under control shRNA conditions, nisin decreased *T. denticola*’s intracellular fluorescence intensity, demonstrating that nisin inhibits *T. denticola* internalization (Fig. 5a and b). These results underscore the essential role of TLR2 in orchestrating the intracellular trafficking of *T. denticola* and nisin. Additionally, to provide direct quantitative evidence supporting TLR2-dependent internalization and intracellular persistence of *T. denticola*, we performed an intracellular survival assay using qPCR to quantify bacterial levels. Briefly, cells under non-transduced control conditions, cells transduced with scrambled shRNA or TLR2 shRNA were infected with wild-type *T. denticola* for 4 h, washed extensively with PBS, and treated with gentamicin for 1 h to eliminate extracellular bacteria. Cells were then either left untreated or exposed to nisin for 24 h. Total DNA was then isolated from both infected and uninfected cells, and intracellular *T. denticola* levels were quantified by qPCR. Detectable intracellular *T. denticola* was observed in control non-transduced conditions and control shRNA conditions, whereas no intracellular bacterial signal was detected in TLR2-suppressed cells, indicating a requirement for TLR2 in bacterial internalization and/or persistence (Supplementary Fig. 2). Moreover, *T. denticola* was not detected in nisin-treated infected cells. Together, these quantitative data complement the confocal microscopy analyses and strengthen the conclusion of TLR2-mediated internalization and downstream bacterial activity.

Given these observations and to assess the unexpected finding from Fig. 4 that TLR2 suppression but not control conditions attenuated dentilisin/PrtP’s gelatinolytic activity, suggesting unique *T. denticola* extracellular and intracellular host-cell dynamics, we further investigated this finding by collecting conditioned media from these cells in Fig. 4 and culturing them anaerobically in OTEM for 7 days. *T. denticola* was detected in control cells but not in TLR2-suppressed cells following gentamicin treatment (Supplementary Fig. 3b), consistent with TLR2-dependent bacterial internalization under these conditions.

### Molecular models of dentilisin/PrtP (PrtP) interaction with pro-MMP2

To investigate potential interactions between MMP2 and dentilisin/PrtP/PrtP), predictive structural models were generated using AlphaFold (Supplementary Fig. 4a–c). The MMP2 structure consisted of four domains, including the catalytic and hemopexin-like regions. For PrtP docking, the subtilisin-like S8 domain was modeled after removing 158 N-terminal residues to represent the mature dentilisin/PrtP/PrtP^74^. Two docking models were selected based on confidence scores and interaction positioning (Supplementary Fig. 5a-d). Model 1 predicted two hydrogen-bond contacts within the PrtP subtilisin domain and the MMP2 hemopexin-like domain: (i) D_dentilisin/PrtP-aa182_ –D_pro-MMP2-aa472_ and (ii) K_dentilisin/PrtP aa407_ –D_pro-MMP2-aa569_. Model 2 predicted a single E _dentilisin/PrtP-aa295_ –K _pro-MMP2-aa327_ interaction between the dentilisin/PrtP/PrtP catalytic region and the MMP2 collagenase domain. These findings suggest that dentilisin/PrtP/PrtP may directly bind functional regions of pro-MMP2, including both the hemopexin-like and catalytic domains. To assess whether nisin interacts with pro-MMP2 or dentilisin/PrtP/PrtP), docking models were generated. A hydrogen bond including K_nisin aa34-_D_dentilisin/PrtP aa240_ was predicted between nisin and PrtP (Supplementary Fig. 6a and b), whereas no such interaction was observed with pro-MMP2 (Supplementary Fig. 6c and d). These findings suggest that nisin may preferentially associate with dentilisin/PrtP under basal conditions. Interactions of the pre-docked dentilisin/PrtP–nisin complex with Pro-MMP2 revealed no detectable interactions, indicating that nisin binding likely induces conformational rearrangements in dentilisin/PrtP that disrupt its hydrogen-bonding interface with Pro-MMP2 (Supplementary Fig. 6e and f).

### Suppression of TLR2 inhibits *T. denticola*-induced MMP2 gene expression as does nisin treatment

**Fig. 6:**
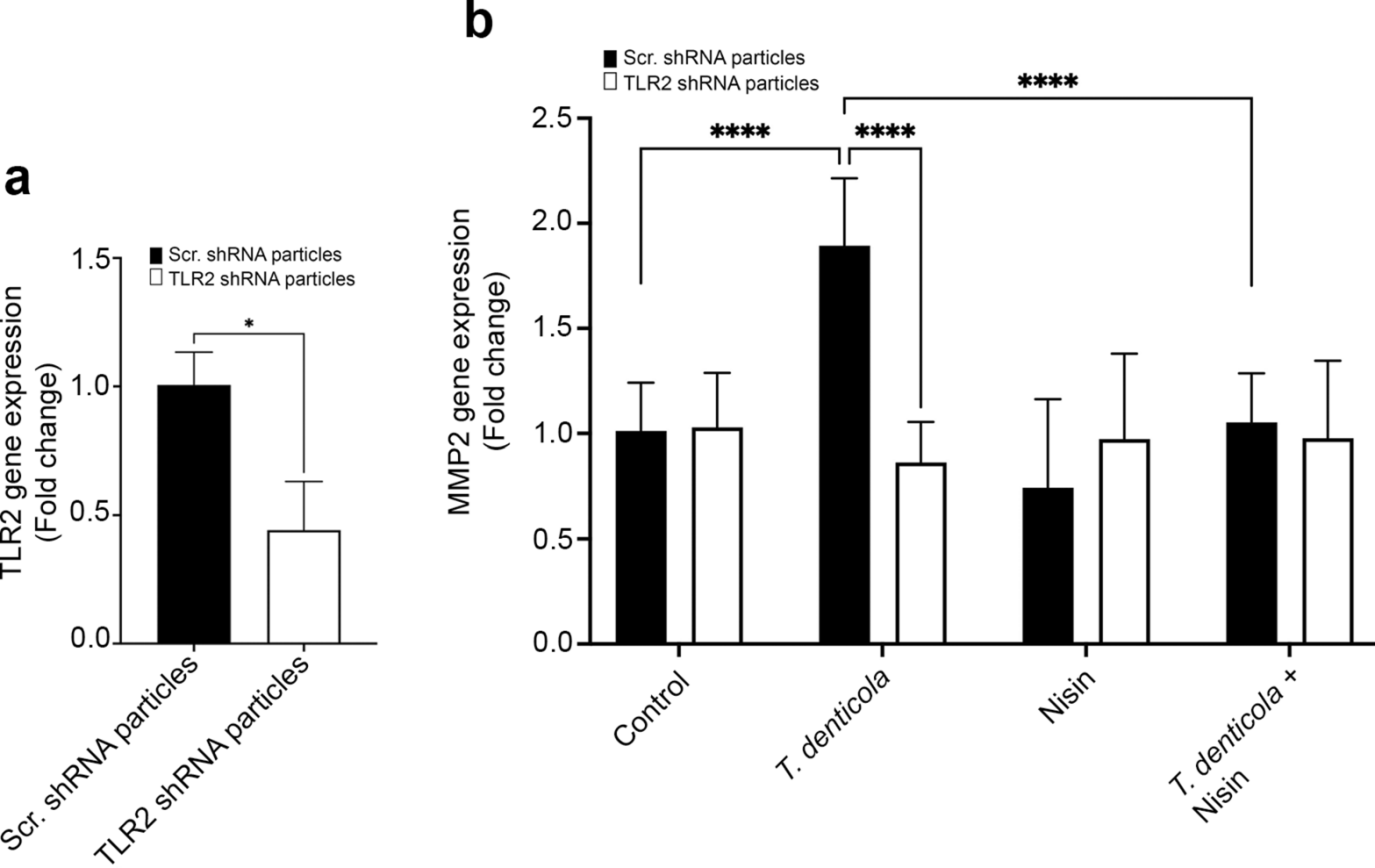
Suppression of TLR2 inhibits *T. denticola*-induced MMP2 gene expression in PDL cells as does nisin treatment. (**a**) Graph showing TLR2 mRNA levels in Scr-shRNA or *TLR2* knockdown cells. (**b**) Graph showing MMP2 mRNA levels in cells exposed to wild-type *T. denticola* (ATCC 35405) (50 MOI) for 2 h, washed three times with PBS, treated with gentamicin (50 µg/ml) for 1 h, washed three times again with PBS, and then treated with nisin (100 µg/ml) for 24 h and analyzed for MMP2 mRNA levels. Data are representative of at least three independent experiments. Intergroup differences were analyzed by the analysis of variance (ANOVA) and Tukey’s post hoc test. *p < 0.05, ** *p* < 0.01, *** *p* < 0.001.

Upon establishing that TLR2 plays a pivotal role in the regulation of *T. denticola*-induced MMP activation, we sought to investigate the degree to which *T. denticola*-induced MMP2 activation and nisin’s effects are mediated at the transcriptional level and via TLR2. Cells transduced with scrambled shRNA or TLR2 shRNA were exposed to wild-type *T. denticola* (ATCC 35405) (50 MOI) for 2 h, washed three times with PBS, treated with gentamicin (50 µg/ml) for 1 h, washed three times again with PBS, and then treated with nisin (100 µg/ml) for 24 h and analyzed for cellular MMP2 mRNA levels. *TLR2* gene suppression was validated via RT-qPCR. Basal TLR2 gene expression was reduced by more than 60% in cells with the TLR2 shRNA construct (Fig. 6a). Suppressing TLR2 gene expression inhibited *T. denticola*-induced MMP2 mRNA expression levels (Fig. 6b). Nisin treatment did not modify these findings, which was not surprising, since nisin was unable to enter the cells in the context of TLR2 suppression as shown in Fig. 5a. However, under scr shRNA conditions, nisin did suppress the *T. denticola*-mediated induction of MMP2 mRNA gene expression (Fig. 5b).

## Discussion

We previously reported that dentilisin/PrtP-triggered TLR2/MyD88 activation upregulated a tissue-destructive program involving increased MMP transcription [27]; however, dentilisin/PrtP’s impact on MMP activation at the protein level and nisin’s modulatory potential in this context had not been determined. In line with previous studies showing that *T. denticola* enhances MMP expression in gingival and periodontal ligament cells through dentilisin/PrtP-dependent mechanisms^14,15,35^, we observed that dentilisin/PrtP-expressing *T. denticola* robustly increased MMP2 activity, whereas a dentilisin/PrtP-deficient mutant strain had no effect, underscoring dentilisin/PrtP’s central role in host matrix degradation. Importantly, nisin treatment significantly mitigated dentilisin/PrtP-induced MMP2 activation while preserving basal MMP2 activity, highlighting its selectivity in modulating pathogenic responses. This study further elucidates TLR2- and nisin-mediated mechanisms that regulate *T. denticola*-driven MMP2 expression in human periodontal ligament cells, emphasizing nisin’s potential as a novel therapeutic approach to modulate host responses during periodontal disease processes.

This study demonstrated that dentilisin/PrtP directly activates recombinant MMP2 in vitro. This observation expands the current understanding of MMP2 regulation, which primarily emphasizes MT1-MMP/TIMP2–dependent activation on the cell surface^75–77^. Similar to other serine proteases, such as plasmin and thrombin, dentilisin/PrtP appears capable of directly cleaving pro–MMP2 to its active form^78^. In addition, previous studies have reported that dentilisin/PrtP interacts with host cell surface receptors, including TLR2, and induces downstream signaling events that can alter protease activity and ECM turnover^32,48,74^. This dual mechanism of dentilisin/PrtP—direct activation of extracellular proteolytic processes and host receptor–mediated signaling—underscores its ability to exacerbate tissue destruction within the periodontal microenvironment.

Using computational modeling, we generated predictive structural models to examine how the *T. denticola* serine protease dentilisin/PrtP (PrtP) engages pro-MMP2 and how the lantibiotic nisin may interfere with this interaction. AlphaFold-derived structures and guided docking revealed two plausible PrtP–pro-MMP2 interfaces: (i) PrtP residues within the peptidase domain contacting the linker between the catalytic and hemopexin-like domains, as well as repeat 3 (R3) of the Pro-MMP2 (Model 1), and (ii) a PrtP glutamic acid (between His258 and Ser447) interacting with a lysine in the catalytic domain of pro-MMP2 (Model 2). These predicted interfaces are consistent with known roles of the hemopexin-like domain in substrate recognition, partner binding, and zymogen activation^79–81^, while also suggesting possible direct engagement near the catalytic cleft. Either binding model could influence pro-MMP2 activation, substrate accessibility, or localization to proteolytically active bacterial surfaces. Notably, nisin preferentially formed a hydrogen bond with PrtP (K_nisin aa34−_D_dentilisin/PrtP aa240_) but not with pro-MMP2. Furthermore, docking of the PrtP–nisin -pro-MMP2 yielded no stable interactions, suggesting that nisin may sterically block or allosterically alter PrtP surfaces required for pro-MMP2 engagement. The gelatin zymography data (Fig. 2) further supports this observation. This modeling suggests that nisin could weaken PrtP-mediated activation of pro-MMP2, thereby affecting extracellular matrix (ECM) remodeling. Given that TIMP-2 physiologically regulates MMP2 by scaffolding pro-MMP2 to MT1-MMP at low concentrations and inhibiting active MMP2 at higher concentrations, our Model 1 prediction is particularly notable^82–84^. PrtP contacts the linker and hemopexin-like domain, overlapping the TIMP-2 binding site. This suggests that PrtP could compete with TIMP-2–mediated recruitment and activation of pro-MMP2, potentially dampening cell surface activation. Alternatively, PrtP might displace inhibitory TIMP-2 from active MMP2, enhancing catalytic activity, though our model favors the former scenario. Finally, we acknowledge limitations inherent to predictive modeling, including reliance on computational structures and docking assumptions, which warrant experimental validation. To address these uncertainties, planned studies will generate site-specific mutations in PrtP residues predicted to interact with MMP2, allowing us to directly test the functional impact of these interactions on pro-MMP2 recruitment and activation, and to clarify the precise molecular mechanism underlying PrtP-mediated modulation of MMP2 activity.

TLR2 is a key sensor for Gram-negative bacterial components in the oral cavity^49^ and is central to host-pathogen signaling pathways^85–88^. We further demonstrate that TLR2 is essential for both *T. denticola*–induced activation of MMP2 and nisin mediated effects. Our findings showing that suppression of TLR2 significantly reduced *T. denticola*-mediated MMP2 activation and *MMP2* gene expression, align with prior reports that dentilisin/PrtP signals via TLR2/MyD88 to regulate inflammatory and degradative pathways^32^. Furthermore, our confocal imaging data revealed that both *T. denticola* and nisin localized to the cytosol in a TLR2-dependent manner, indicating that TLR2 receptor-mediated internalization and intracellular trafficking processes are involved. This is in line with evidence that TLR2 ligands can be internalized to endosomal compartments where signaling continues^89^. While the internalization of *T. denticola* into host cells has been documented^15,74,90,91^, our study demonstrates that nisin is also internalized and uptake is similarly dependent on TLR2 mechanisms, highlighting the important concept that nisin can target intracellular pathogens that enter and reside inside host cells and also mitigate gene expression process activated by pathogens. Importantly, nisin was able to further decrease *T. denticola* internalization.

Although *T. denticola* is a strict obligate anaerobe, previous studies suggest that it can persist transiently within host cells, where hypoxic or microaerophilic intracellular niches may permit short-term survival. Such environments may partially protect anaerobic bacteria from oxygen exposure, allowing *T. denticola* to remain metabolically active long enough to initiate host signaling^92^. However, sustained intracellular viability under experimental conditions is likely limited, indicating that prolonged bacterial survival may not be required for the observed host responses. Importantly, dentilisin, a major outer-membrane–associated protease and virulence factor of *T. denticola*, is known to exert potent biological effects independent of bacterial viability, including modulation of host signaling, degradation of extracellular matrix components, disruption of epithelial barrier integrity, and activation of inflammatory pathways^48^. Collectively, these findings suggest that the observed effects are mediated by both transient intracellular *T. denticola* and dentilisin activity, with dentilisin likely playing a dominant role. Notably, our intracellular viability assay demonstrated that *T. denticola* survived for at least 72 h, supporting a potential contribution of intracellular bacteria to the initiation of host responses.

Nisin’s dual function as an antimicrobial and host-modulatory agent enhances its potential as a therapeutic adjunct. In addition to its bactericidal efficacy against oral pathogens, recent research has demonstrated that nisin downregulates proinflammatory pathways in mammalian cells, modulates MAPK/AKT signaling, and mitigates host responses in animal models of inflammatory disease^55,63,93^. Within the context of periodontal disease, the use of nisin-producing *Lactococcus lactis* or treatment with nisin directly can decrease bacterial load, inflammation, and alveolar bone loss^54,94^. Our findings further enhance these observations by demonstrating that nisin can inhibit dentilisin/PrtP-driven activation of MMP2, thereby directly addressing a pivotal mechanism involved in connective tissue destruction in periodontitis. Importantly, nisin also attenuates *T. denticola* host cell internalization. Thus, nisin may serve as a multi-modal therapeutic that mediates antimicrobial effects at different levels; namely by directly inhibiting bacterial survival, abrogating virulence factors like *T. denticola* dentilisin/PrtP activity, and attenuating bacterial entry into host cells and thereby suppressing harmful intracellular signaling.

The combined data from this study further highlight unique microbe-host cell dynamics indicating that *T. denticola* gains entry into host cells, but it may also exit cells under favorable conditions. Its entry into host cells enables it to evade the host immune response, as has been shown for other pathogenic microbes^95–97^. The ability of *T. denticola* to enter host cells is in agreement with other published data^90–92^, and furthermore, other pathogenic bacteria, such as *Porphyromonas gingivalis (p. gingivalis)*, are also known to enter host cells^98–100^. Pathogens are also known to exit host cells via a variety of mechanisms that include lytic and non-lytic mechanisms (budding, extracellular vesicles, programmed cell death)^101–104^. Further studies are needed to explore *T. denticola’s* ability to exit host cells, and from these current observations it appears to mediate its exit via non-lytic processes.

In summary, this study emphasizes dentilisin/PrtP as a principal driver of MMP2 activation in PDL cells and recognizes nisin as a selective modulator that interferes with both enzymatic and receptor-mediated activation pathways. By targeting this vital axis of periodontal pathogenesis, nisin demonstrates potential as a useful adjunct to conventional periodontal therapy.

## Materials and methods

### PDL cell culture

Human periodontal ligament (PDL) cells (Cat #2630, ScienCell Research Laboratories, Carlsbad, CA) were maintained in MEM media containing 10% fetal bovine serum and 1% P/S at 37 °C in 5% CO_2_ as described earlier^32^.

### Anaerobic bacterial cultures

*T. denticola* (Td-WT) (Cat #35405, ATCC, Manassas, VA) and *T. denticola* CF522 (Td-MT), its isogenic dentilisin/PrtP-deficient mutant^105^ were cultured in Oral Treponeme Enrichment Broth (OTEB; Cat #AS-603, Anaerobe Systems, Morgan Hill, CA) at 37 °C in an anaerobic chamber (Cat #BAA30023, Bactron300, LabRepCo) supplied with 5% H₂, 10% CO₂, and 85% N₂ mixed gases.

### Purification of *T. denticola* dentilisin/PrtP

The dentilisin/PrtP protease complex was purified from the detergent phase of Triton X-114 extracts of an isogenic *T. denticola* mutant that lacks Msp^106^, the dominant outer membrane protein as previously described^107^.

### Nisin Z preparation

An ultra-pure (> 95%) food-grade preparation of nisin Z (Nisin Z; Handary S.A., Brussels, Belgium) was dissolved in sterile Milli-Q water to a stock concentration of 5 mg/mL, filtered through a 0.22 μm syringe filter, and stored at 4 °C for a maximum of 5 days for use in experiments.

### *T. denticola*-PDL cell interactions and nisin treatment

To characterize the specific roles of *T. denticola* in microbe–host interactions, PDL cells were grown to ~ 80% confluence (1.2 × 10^6^ cells per 6-cm dish) and challenged with *T. denticola* at a multiplicity of infection (MOI) of 50 in serum- and antibiotic-free medium for 2 h, as described previously^14,108,109^. After a two-hour challenge with *T. denticola*, PDL cells were washed with PBS three times, treated with 100 µg/ml nisin in serum-free- and antibiotic-free medium for 24 h, then conditioned media was collected from these cells and analyzed for MMP2 activity via gelatin zymography. The protein concentration of the conditioned media was calculated for each sample using the Pierce BCA Protein Assay kit according to the manufacturer’s instructions (Cat #23225, Thermo Fisher Scientific, Waltham, MA). Equal amounts of protein (50 µg) from each conditioned media sample were mixed with 4X sample buffer (Cat #1610747, Bio-Rad, Hercules, CA (15 µl) and loaded onto 10% gelatin-containing SDS-PAGE gels (Cat #ZY00100BOX, Thermo Fisher scientific, Novex TM 10% zymogram plus (Gelatin) protein gels). Gelatin zymography was conducted as previously described^15,35^.

### In vitro assay for dentilisin/PrtP-mediated MMP2 activation

For in vitro MMP2 activation, different concentrations of purified dentilisin/PrtP (20, 40 or 80 ng)^107,110^ and pro-MMP2 (20 ng) (Cat #902-MP-010; R&D Systems, Minneapolis, MN) diluted in reaction buffer (TCNB, 50 mM Tris, 10 mM CaCl_2_, 150 mM NaCl, 0.05% (w/v) Brij 35, pH 7.5) were mixed and incubated for 30 min at room temperature, then mixed with 4X sample buffer (Cat #1610747, Bio-Rad, Hercules, CA) and loaded onto 10% gelatin-containing SDS-PAGE gels (Cat #ZY00100BOX, Thermo Fisher scientific, Novex TM 10% zymogram plus (Gelatin) protein gels).

### Gelatin zymography

Gelatin zymography was conducted as previously described^15,35,107^. Briefly, after electrophoresis, SDS was removed from the gels by washing them in renaturing buffer (Cat #LC2670, Novex, Waltham, MA) for 30 min, then the gels were placed in developing buffer (Cat #LC2671, Novex, Waltham, MA) for 30 min. The developing buffer was changed, and gels were incubated at 37 °C for 16 h, then stained with SimplyBlue SafeStain (Cat #465034, Invitrogen, Waltham, MA) for 3 h, and de-stained in 10% methanol and 5% acetic acid until clear bands appeared against a blue background. Zymogram images were captured using ChemiDoc MP image system (Bio-Rad, Hercules, CA), and the band intensity for pro- and active-MMP2 expression was quantified using ImageJ software.

### 3D structural modeling and computational analysis of protein interactions

Amino acid sequences of pro-MMP2 and dentilisin/PrtP (PrtP) were obtained from NCBI, while the nisin sequence was retrieved from published data^111^. Three-dimensional structure prediction and domain annotation were performed using AlphaFold^112^ and ExPASy PROSITE^113^. Homology models were generated with SWISS-MODEL^114^, and protein–protein docking was conducted with HDOCK^115^, considering models with confidence scores ≥ 0.7 as high-confidence. Predicted and docked structures were visualized and analyzed using UCSF ChimeraX^116^.

### Stable suppression of TLR2

PDL cells were grown to ~ 80% confluence (3.0 × 10^5^ cells per 3.5-cm dish) and transduced with short hairpin RNA (shRNA) lentiviral particles specific for TLR2 (30 µl, sc-40256-V, Santa Cruz Biotechnology, Dallas, TX) or scrambled-shRNA (30 µl, SC-108080; Santa Cruz Biotechnology) lentiviral particles in 0.5 mL of serum-free media containing polybrene then selected in 5 µg/mL puromycin (sc-108071; Santa Cruz Biotechnology) for an additional 10 days^32^. Surviving cell colonies were picked and propagated before testing for TLR2 expression using Western blot analyses.

### Western blotting

Western blot analyses to detect the levels of TLR expression were performed using an anti-TLR2 antibody (ab213676, Abcam, Cambridge, MA), followed by a horseradish peroxidase-conjugated anti-mouse antibody (SC-2005, Santa Cruz Biotechnology). Blots were then developed with the ECL-plus detection system (Cat #34577, Thermo Fisher Scientific, Waltham, MA). To evaluate the samples for equal protein loading, membranes were stripped and re-probed with an anti-β-actin antibody (SC-1615, Santa Cruz Biotechnology, Dallas, TX).

### Confocal immunofluorescence microscopy

PDL cells were grown to ~ 80% confluence (3.0 × 10^5^ cells per 3.5-cm dish) then cells were exposed to Syto 9-stained-(1:1000 dilution, S34854, Life Technologies, Waltham, MA) *T. denticola* (50 MOI) for 4 h, treated with gentamicin (50 µg/ml) (Cat #15750060, Thermo Fisher Scientific, Waltham, MA) for 1 h to remove any remaining extracellular *T. denticola*, then treated with nisin (100 µg/ml) for 24 h. After treatment, cells were washed with PBS, fixed with 4% paraformaldehyde, and permeabilized with 0.2% Triton X-100. To block for non-specific antibody binding, cells were treated with 10% serum before incubation with a nisin primary antibody overnight. The custom polyclonal affinity-purified rabbit nisin antibody against nisin (proprietary to Kapila lab) was produced by co-immunizing rabbits with nisin peptide sequences (Pacific Immunology, Ramona, CA). The sequences used for the nisin antibody production were ITSISLC, KTGALMGC, CNMKTAT, and CSIHVSK. Next, cells were washed three times with PBS, incubated in a secondary antibody conjugated to Alexa Fluor 647 (Cat #A31573, Life Technologies, Waltham, MA) for 30 min at 37˚C, and then stained with DAPI (Cat #28718-90-3, Sigma, St. Louis, MO) for nuclear staining. Finally, cells were washed with PBS and mounted with Faramount media (Cat #S3025, Dako, Carpinteria, CA). Confocal imaging was performed using a TCS SP8 X Confocal Laser Scanning Microscope (Leica, Wetzlar, Germany) equipped with a 63X oil-immersion objective (NA 1.4, FN25, APO, 506350). For each group, images were collected from five independent fields at the plane of maximum nuclear width. Fluorescence acquisition parameters were optimized and fixed across all samples as follows: SYTO 9-stained *T. denticola* (excitation 483 nm, emission 503 nm), DAPI (excitation 350 nm, emission 470 nm), and nisin (excitation 650 nm, emission 665 nm). Image acquisition was performed using Leica Application Suite X (LAS X) software, and subsequent fluorescence intensity measurements for *T. denticola* and nisin were quantified using FIJI (ImageJ)^117^.

### Intracellular *T. denticola* quantification by quantitative PCR (qPCR)^118^

PDL cells under control non-transduced conditions, cells transduced with scrambled shRNA or TLR2 shRNA were exposed to wild-type *T. denticola* (35405, 50 MOI) for 4 h, washed three times with PBS, treated with gentamicin (50 µg/ml) for 1 h, washed again, and then treated with nisin (100 µg/ml) for 24 h. Cells were harvested and DNA was isolated cells using the QIAamp DNA mini kit (Cat #51304, Qiagen, Germantown, MD) and *T. denticola* levels were quantified by qPCR. After harvest, total DNA was extracted from infected and non-infected cells using the QIAamp DNA Mini Kit (Qiagen, USA) according to the manufacturer’s instructions. DNA concentration and purity were assessed using a NanoDrop One spectrophotometer (Thermo Fisher Scientific, USA), and samples were stored at − 80 °C until further analysis. Intracellular *T. denticola* levels were quantified by qPCR. For each reaction, 400 ng of template DNA was combined with 10 µL of TaqMan Fast Advanced Master Mix and 1 µL of *T. denticola*–specific primers and probe (TaqMan Gene Expression Assay, Cat# Ba07922667_s1; Thermo Fisher Scientific, USA). qPCR was performed using a QuantStudio 3 Real-Time PCR System (Thermo Fisher Scientific, USA) following the manufacturer’s protocol. Cycle threshold (Ct) values were determined using QuantStudio Design and Analysis Software (v1.6.1; Thermo Fisher Scientific, USA), with a cutoff of 40 amplification cycles used to define detection.

### Quantitative RT-PCR (qRT-PCR)

RNA was isolated from PDL cells using the RNeasy Mini kit (Cat #74104, Qiagen, Germantown, MD) per the manufacturer’s instructions. RNA was then reverse transcribed to cDNA using SuperScript VILO cDNA Synthesis Kit (Cat#11754- 050, Invitrogen, Waltham, MA) and amplified by qRT-PCR using gene-specific primers for TLR2 (Cat #Hs01872448_−_s1, Life Technologies, Waltham, MA) or MMP2 (Cat #Hs01548727_−_m1, Life Technologies). Relative gene expression levels were plotted as fold change compared to untreated or negative controls. The 2^−^ΔΔCT method was used to determine the relative change in gene expression, normalized against GAPDH (Cat #Mm99999915_g1, Life Technologies, ).

### Statistical analysis

All experiments were performed with three independent biological replicates (*n* = 3). Intergroup differences were analyzed by the analysis of variance (ANOVA) and Tukey’s post hoc test. *p < 0.05, ** *p* < 0.01, *** *p* < 0.001, **** *p* < 0.0001.

## Supplementary Information

Below is the link to the electronic supplementary material.

Supplementary Material 1

Supplementary Material 2

## Data Availability

All data generated or analyzed during this study are included in this published article and its supplementary information files.
