# Supplementary material for: Nisin bacteriocin blocks T. denticola-triggered MMP2 activation and pathogen internalization via TLR2
Source: Sci Rep. 2026 Mar 11;16:13085. doi: 10.1038/s41598-026-43673-8 (PMC13100033; doi:10.1038/s41598-026-43673-8)
Supplement: Supplementary file 1 — Supplementary Material 1 [file 41598_2026_43673_MOESM1_ESM.pptx]

## Slide 1
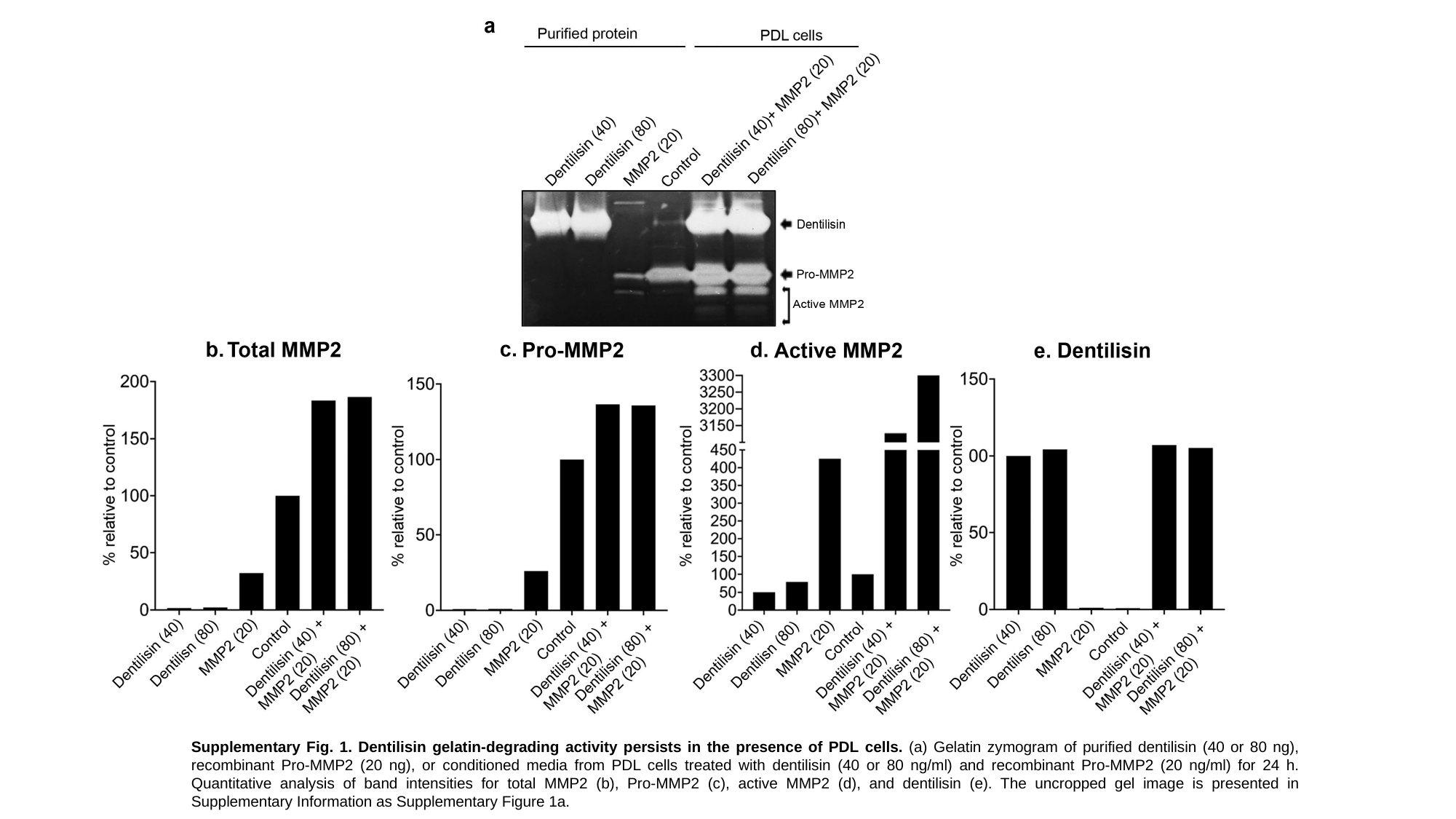

Supplementary Fig. 1. Dentilisin gelatin-degrading activity persists in the presence of PDL cells. (a) Gelatin zymogram of purified dentilisin (40 or 80 ng), recombinant Pro-MMP2 (20 ng), or conditioned media from PDL cells treated with dentilisin (40 or 80 ng/ml) and recombinant Pro-MMP2 (20 ng/ml) for 24 h. Quantitative analysis of band intensities for total MMP2 (b), Pro-MMP2 (c), active MMP2 (d), and dentilisin (e). The uncropped gel image is presented in Supplementary Information as Supplementary Figure 1a.

## Slide 2
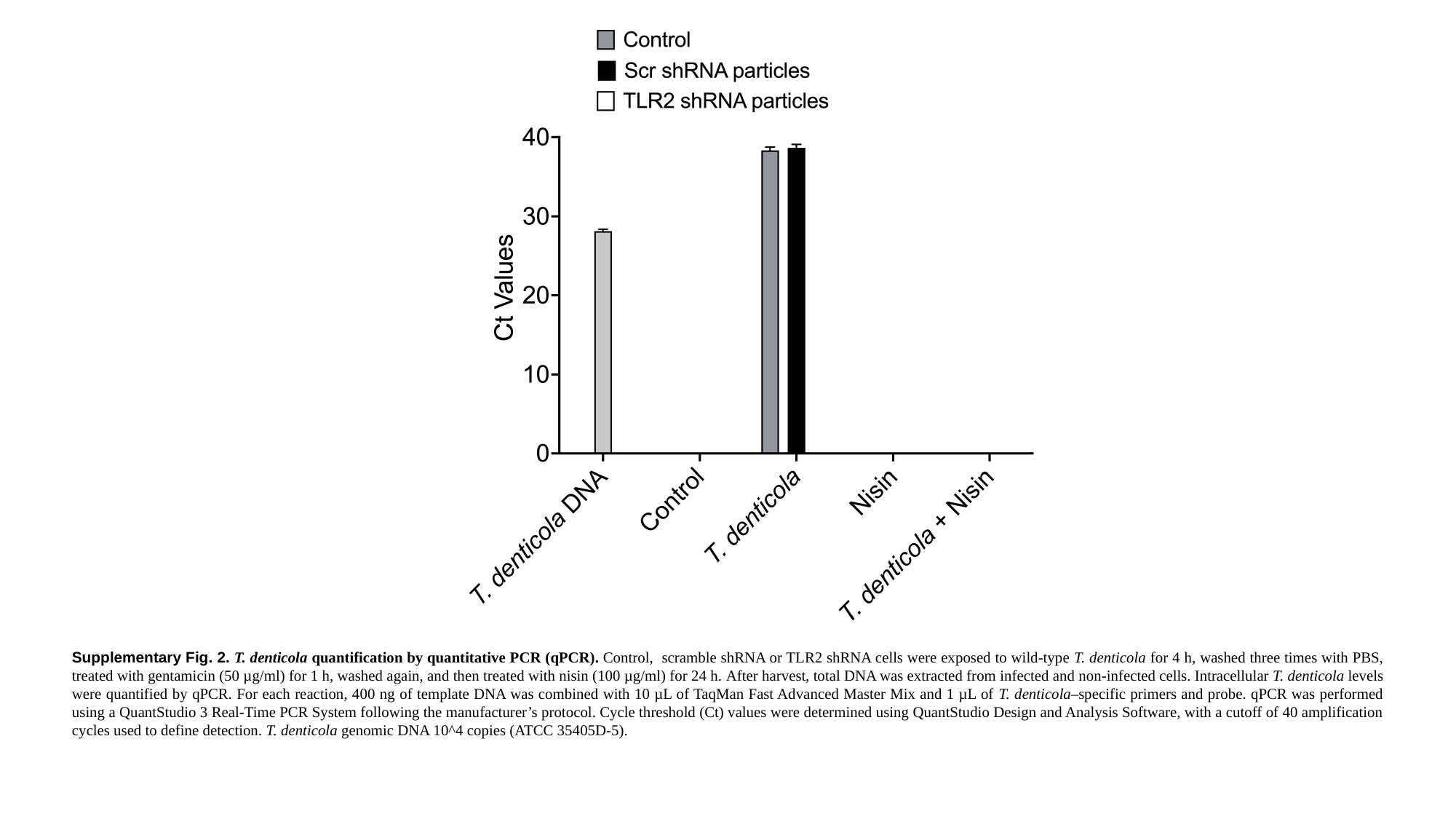

Supplementary Fig. 2. T. denticola quantification by quantitative PCR (qPCR). Control, scramble shRNA or TLR2 shRNA cells were exposed to wild-type T. denticola for 4 h, washed three times with PBS, treated with gentamicin (50 µg/ml) for 1 h, washed again, and then treated with nisin (100 µg/ml) for 24 h. After harvest, total DNA was extracted from infected and non-infected cells. Intracellular T. denticola levels were quantified by qPCR. For each reaction, 400 ng of template DNA was combined with 10 µL of TaqMan Fast Advanced Master Mix and 1 µL of T. denticola–specific primers and probe. qPCR was performed using a QuantStudio 3 Real-Time PCR System following the manufacturer’s protocol. Cycle threshold (Ct) values were determined using QuantStudio Design and Analysis Software, with a cutoff of 40 amplification cycles used to define detection. T. denticola genomic DNA 10^4 copies (ATCC 35405D-5).

## Slide 3
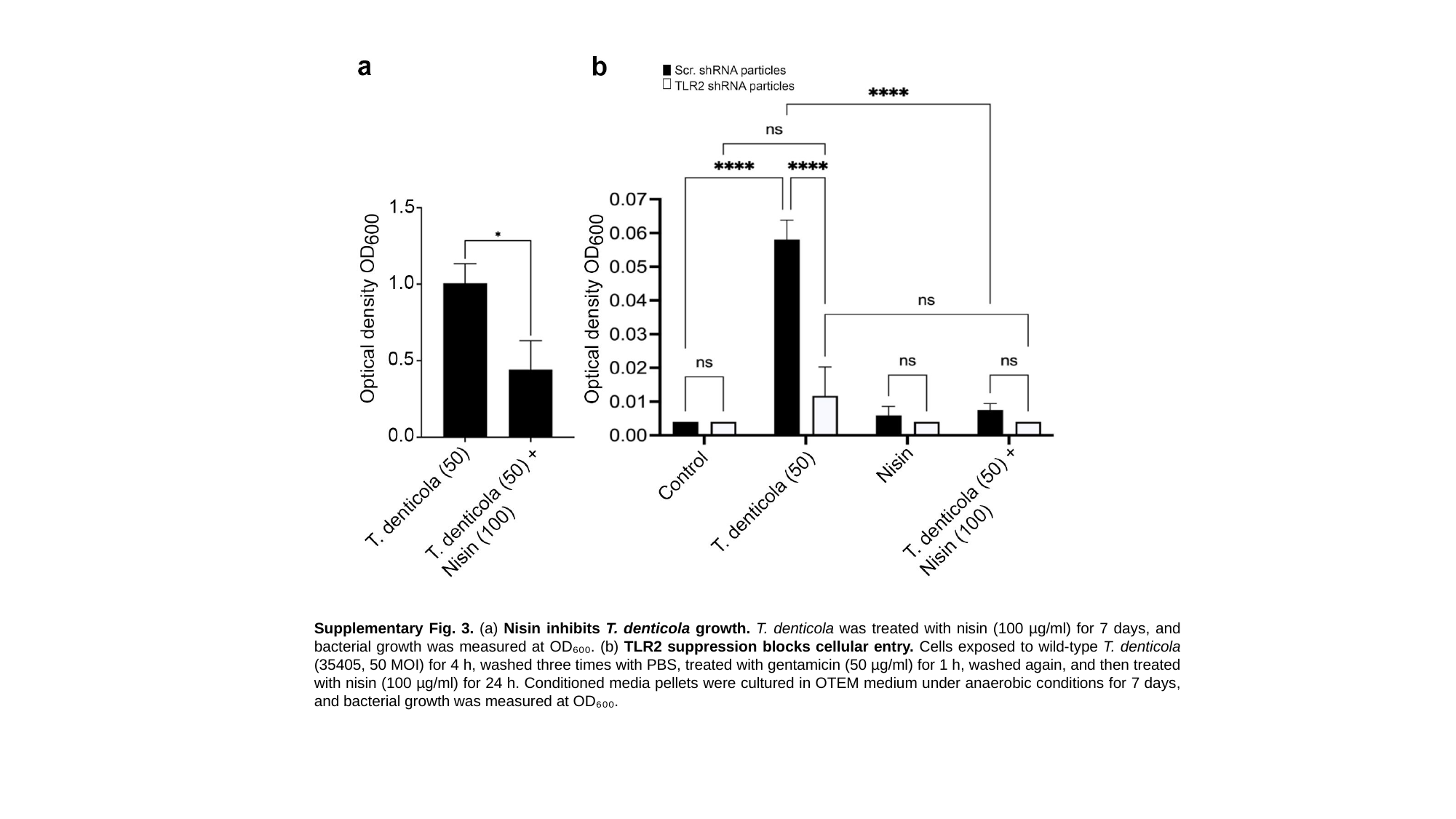

Supplementary Fig. 3. (a) Nisin inhibits T. denticola growth. T. denticola was treated with nisin (100 µg/ml) for 7 days, and bacterial growth was measured at OD₆₀₀. (b) TLR2 suppression blocks cellular entry. Cells exposed to wild-type T. denticola (35405, 50 MOI) for 4 h, washed three times with PBS, treated with gentamicin (50 µg/ml) for 1 h, washed again, and then treated with nisin (100 µg/ml) for 24 h. Conditioned media pellets were cultured in OTEM medium under anaerobic conditions for 7 days, and bacterial growth was measured at OD₆₀₀.

## Slide 4
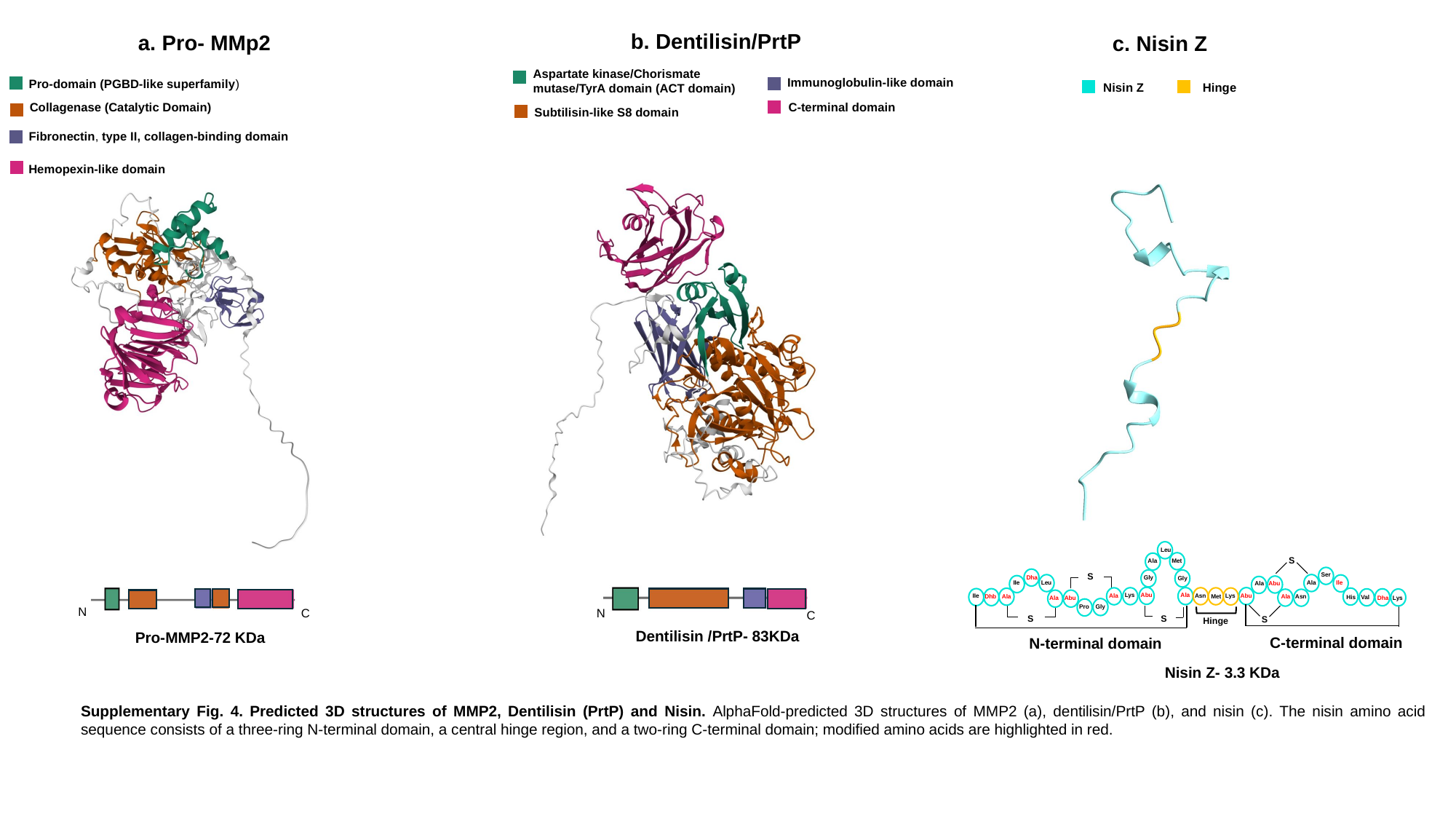

b. Dentilisin/PrtP
a. Pro- MMp2
c. Nisin Z
Aspartate kinase/Chorismate mutase/TyrA domain (ACT domain)
Immunoglobulin-like domain
Pro-domain (PGBD-like superfamily)
Nisin Z
Hinge
C-terminal domain
Collagenase (Catalytic Domain)
Subtilisin-like S8 domain
Fibronectin, type II, collagen-binding domain
Hemopexin-like domain
Leu
S
Ala
Met
S
Ser
Dha
Gly
Gly
IIe
Ile
Leu
Ala
Abu
Ala
N
C
Pro-MMP2-72 KDa
Ala
Lys
Abu
Ala
Asn
Lys
Abu
IIe
Dhb
Ala
Met
Asn
Ala
His
Val
Lys
Ala
Abu
Dha
Pro
Gly
N
C
S
S
S
Hinge
Dentilisin /PrtP- 83KDa
C-terminal domain
N-terminal domain
Nisin Z- 3.3 KDa
Supplementary Fig. 4. Predicted 3D structures of MMP2, Dentilisin (PrtP) and Nisin. AlphaFold-predicted 3D structures of MMP2 (a), dentilisin/PrtP (b), and nisin (c). The nisin amino acid sequence consists of a three-ring N-terminal domain, a central hinge region, and a two-ring C-terminal domain; modified amino acids are highlighted in red.

## Slide 5
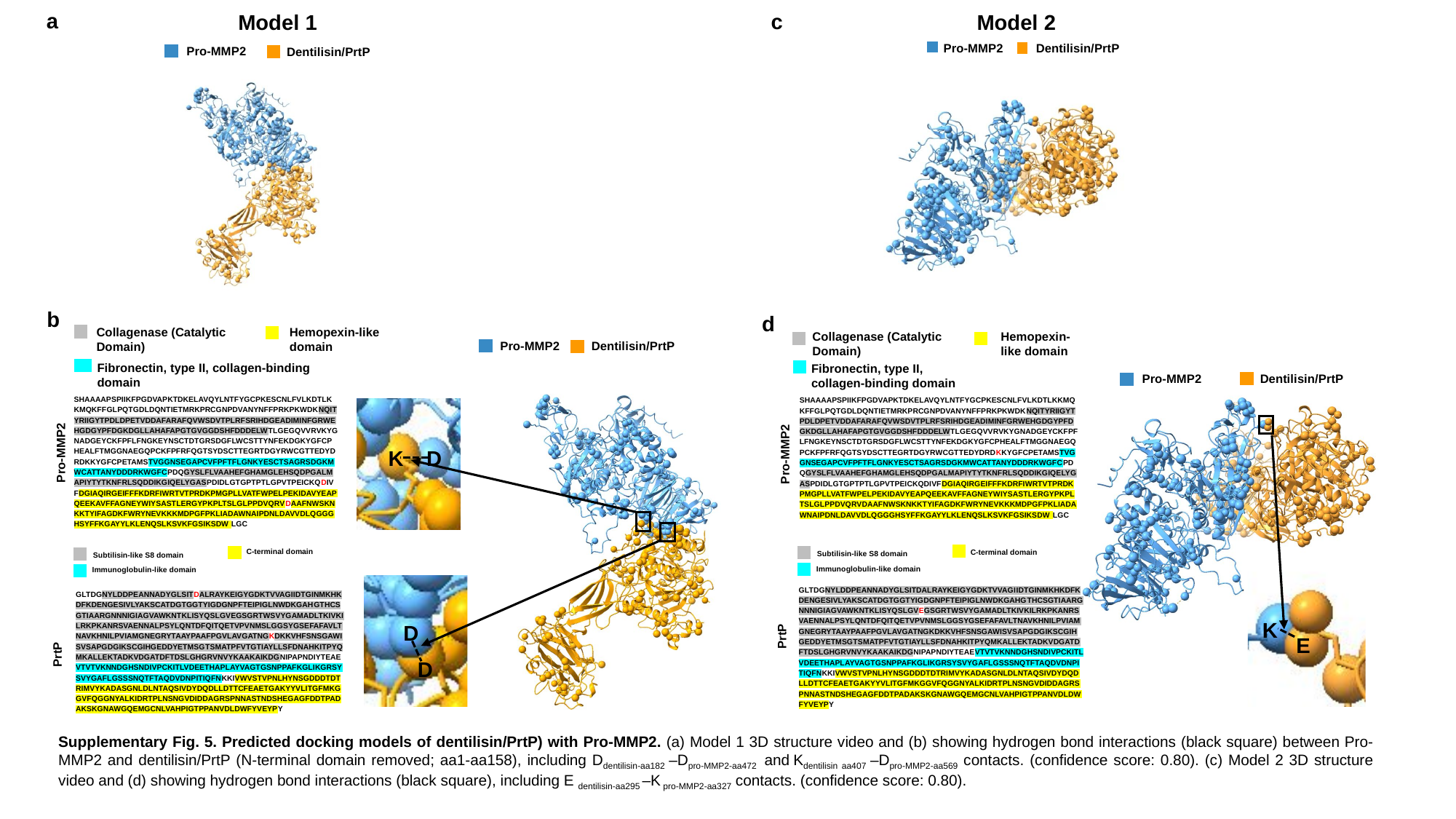

a
c
Model 2
Model 1
Pro-MMP2
Dentilisin/PrtP
Pro-MMP2
Dentilisin/PrtP
b
d
Collagenase (Catalytic Domain)
Hemopexin-like domain
Collagenase (Catalytic Domain)
Hemopexin-like domain
Pro-MMP2
Dentilisin/PrtP
Fibronectin, type II, collagen-binding domain
Fibronectin, type II, collagen-binding domain
Dentilisin/PrtP
Pro-MMP2
K
E
SHAAAAPSPIIKFPGDVAPKTDKELAVQYLNTFYGCPKESCNLFVLKDTLKKMQKFFGLPQTGDLDQNTIETMRKPRCGNPDVANYNFFPRKPKWDKNQITYRIIGYTPDLDPETVDDAFARAFQVWSDVTPLRFSRIHDGEADIMINFGRWEHGDGYPFDGKDGLLAHAFAPGTGVGGDSHFDDDELWTLGEGQVVRVKYGNADGEYCKFPFLFNGKEYNSCTDTGRSDGFLWCSTTYNFEKDGKYGFCPHEALFTMGGNAEGQPCKFPFRFQGTSYDSCTTEGRTDGYRWCGTTEDYDRDKKYGFCPETAMSTVGGNSEGAPCVFPFTFLGNKYESCTSAGRSDGKMWCATTANYDDDRKWGFCPDQGYSLFLVAAHEFGHAMGLEHSQDPGALMAPIYTYTKNFRLSQDDIKGIQELYGASPDIDLGTGPTPTLGPVTPEICKQDIVFDGIAQIRGEIFFFKDRFIWRTVTPRDKPMGPLLVATFWPELPEKIDAVYEAPQEEKAVFFAGNEYWIYSASTLERGYPKPLTSLGLPPDVQRVDAAFNWSKNKKTYIFAGDKFWRYNEVKKKMDPGFPKLIADAWNAIPDNLDAVVDLQGGGHSYFFKGAYYLKLENQSLKSVKFGSIKSDW LGC
SHAAAAPSPIIKFPGDVAPKTDKELAVQYLNTFYGCPKESCNLFVLKDTLKKMQKFFGLPQTGDLDQNTIETMRKPRCGNPDVANYNFFPRKPKWDKNQITYRIIGYTPDLDPETVDDAFARAFQVWSDVTPLRFSRIHDGEADIMINFGRWEHGDGYPFDGKDGLLAHAFAPGTGVGGDSHFDDDELWTLGEGQVVRVKYGNADGEYCKFPFLFNGKEYNSCTDTGRSDGFLWCSTTYNFEKDGKYGFCPHEALFTMGGNAEGQPCKFPFRFQGTSYDSCTTEGRTDGYRWCGTTEDYDRDKKYGFCPETAMSTVGGNSEGAPCVFPFTFLGNKYESCTSAGRSDGKMWCATTANYDDDRKWGFCPDQGYSLFLVAAHEFGHAMGLEHSQDPGALMAPIYTYTKNFRLSQDDIKGIQELYGASPDIDLGTGPTPTLGPVTPEICKQDIVFDGIAQIRGEIFFFKDRFIWRTVTPRDKPMGPLLVATFWPELPEKIDAVYEAPQEEKAVFFAGNEYWIYSASTLERGYPKPLTSLGLPPDVQRVDAAFNWSKNKKTYIFAGDKFWRYNEVKKKMDPGFPKLIADAWNAIPDNLDAVVDLQGGGHSYFFKGAYYLKLENQSLKSVKFGSIKSDW LGC
K
 D
Pro-MMP2
Pro-MMP2
C-terminal domain
C-terminal domain
Subtilisin-like S8 domain
Subtilisin-like S8 domain
Immunoglobulin-like domain
Immunoglobulin-like domain
D
D
GLTDGNYLDDPEANNADYGLSITDALRAYKEIGYGDKTVVAGIIDTGINMKHKDFKDENGESIVLYAKSCATDGTGGTYIGDGNPFTEIPIGLNWDKGAHGTHCSGTIAARGNNNIGIAGVAWKNTKLISYQSLGVEGSGRTWSVYGAMADLTKIVKILRKPKANRSVAENNALPSYLQNTDFQITQETVPVNMSLGGSYGSEFAFAVLTNAVKHNILPVIAMGNEGRYTAAYPAAFPGVLAVGATNGKDKKVHFSNSGAWISVSAPGDGIKSCGIHGEDDYETMSGTSMATPFVTGTIAYLLSFDNAHKITPYQMKALLEKTADKVDGATDFTDSLGHGRVNVYKAAKAIKDGNIPAPNDIYTEAEVTVTVKNNDGHSNDIVPCKITLVDEETHAPLAYVAGTGSNPPAFKGLIKGRSYSVYGAFLGSSSNQTFTAQDVDNPITIQFNKKIVWVSTVPNLHYNSGDDDTDTRIMVYKADASGNLDLNTAQSIVDYDQDLLDTTCFEAETGAKYYVLITGFMKGGVFQGGNYALKIDRTPLNSNGVDIDDAGRSPNNASTNDSHEGAGFDDTPADAKSKGNAWGQEMGCNLVAHPIGTPPANVDLDWFYVEYPY
GLTDGNYLDDPEANNADYGLSITDALRAYKEIGYGDKTVVAGIIDTGINMKHKDFKDENGESIVLYAKSCATDGTGGTYIGDGNPFTEIPIGLNWDKGAHGTHCSGTIAARGNNNIGIAGVAWKNTKLISYQSLGVEGSGRTWSVYGAMADLTKIVKILRKPKANRSVAENNALPSYLQNTDFQITQETVPVNMSLGGSYGSEFAFAVLTNAVKHNILPVIAMGNEGRYTAAYPAAFPGVLAVGATNGKDKKVHFSNSGAWISVSAPGDGIKSCGIHGEDDYETMSGTSMATPFVTGTIAYLLSFDNAHKITPYQMKALLEKTADKVDGATDFTDSLGHGRVNVYKAAKAIKDGNIPAPNDIYTEAEVTVTVKNNDGHSNDIVPCKITLVDEETHAPLAYVAGTGSNPPAFKGLIKGRSYSVYGAFLGSSSNQTFTAQDVDNPITIQFNKKIVWVSTVPNLHYNSGDDDTDTRIMVYKADASGNLDLNTAQSIVDYDQDLLDTTCFEAETGAKYYVLITGFMKGGVFQGGNYALKIDRTPLNSNGVDIDDAGRSPNNASTNDSHEGAGFDDTPADAKSKGNAWGQEMGCNLVAHPIGTPPANVDLDWFYVEYPY
PrtP
PrtP
Supplementary Fig. 5. Predicted docking models of dentilisin/PrtP) with Pro-MMP2. (a) Model 1 3D structure video and (b) showing hydrogen bond interactions (black square) between Pro-MMP2 and dentilisin/PrtP (N-terminal domain removed; aa1-aa158), including Ddentilisin-aa182 –Dpro-MMP2-aa472 and Kdentilisin aa407 –Dpro-MMP2-aa569 contacts. (confidence score: 0.80). (c) Model 2 3D structure video and (d) showing hydrogen bond interactions (black square), including E dentilisin-aa295 –K pro-MMP2-aa327 contacts. (confidence score: 0.80).

## Slide 6
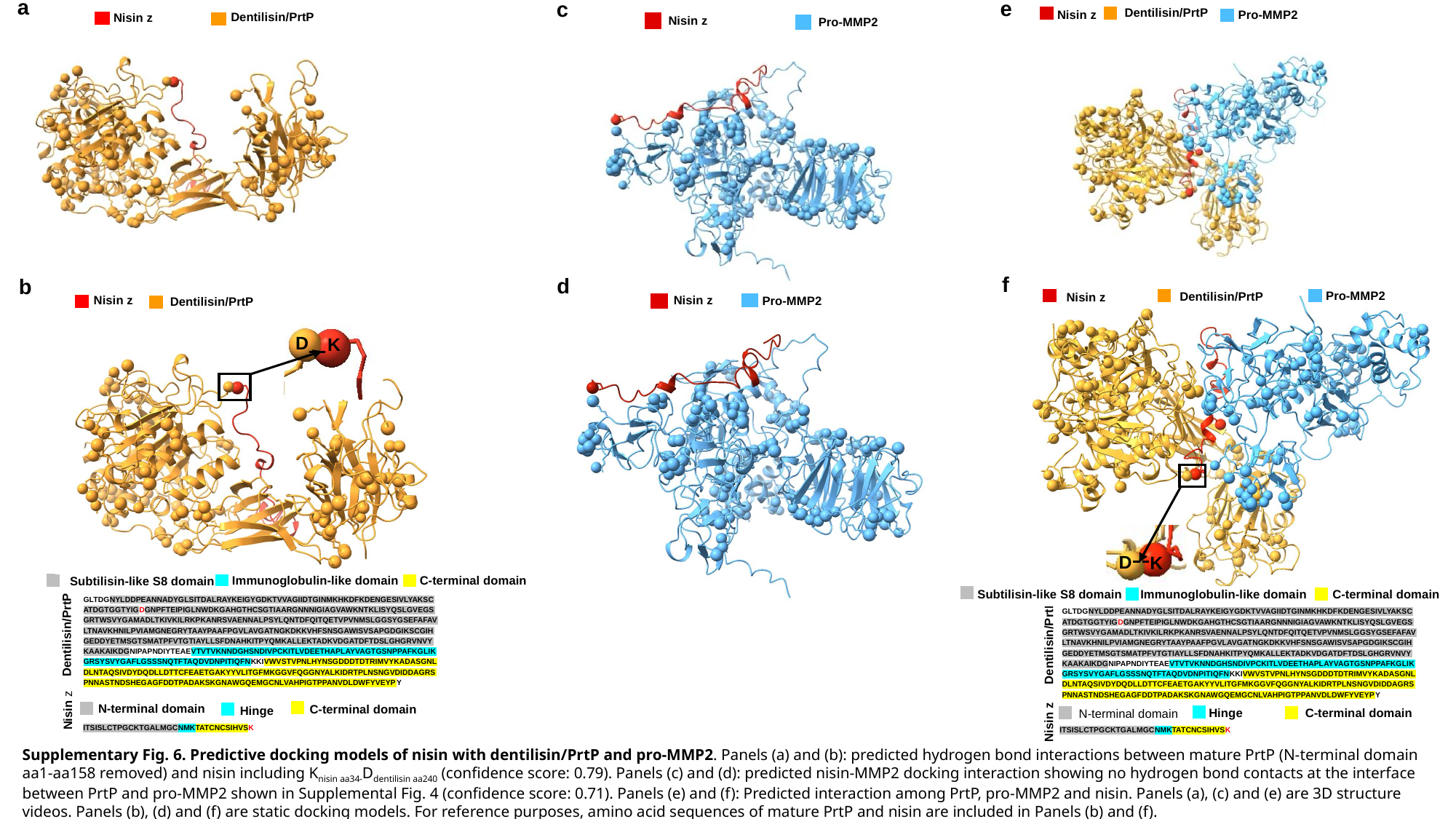

a
e
c
Dentilisin/PrtP
Nisin z
Pro-MMP2
Dentilisin/PrtP
Nisin z
Nisin z
Pro-MMP2
f
d
b
Pro-MMP2
Dentilisin/PrtP
Nisin z
Nisin z
Nisin z
Pro-MMP2
Dentilisin/PrtP
D
K
D
K
C-terminal domain
Immunoglobulin-like domain
Subtilisin-like S8 domain
Subtilisin-like S8 domain
C-terminal domain
Immunoglobulin-like domain
GLTDGNYLDDPEANNADYGLSITDALRAYKEIGYGDKTVVAGIIDTGINMKHKDFKDENGESIVLYAKSCATDGTGGTYIGDGNPFTEIPIGLNWDKGAHGTHCSGTIAARGNNNIGIAGVAWKNTKLISYQSLGVEGSGRTWSVYGAMADLTKIVKILRKPKANRSVAENNALPSYLQNTDFQITQETVPVNMSLGGSYGSEFAFAVLTNAVKHNILPVIAMGNEGRYTAAYPAAFPGVLAVGATNGKDKKVHFSNSGAWISVSAPGDGIKSCGIHGEDDYETMSGTSMATPFVTGTIAYLLSFDNAHKITPYQMKALLEKTADKVDGATDFTDSLGHGRVNVYKAAKAIKDGNIPAPNDIYTEAEVTVTVKNNDGHSNDIVPCKITLVDEETHAPLAYVAGTGSNPPAFKGLIKGRSYSVYGAFLGSSSNQTFTAQDVDNPITIQFNKKIVWVSTVPNLHYNSGDDDTDTRIMVYKADASGNLDLNTAQSIVDYDQDLLDTTCFEAETGAKYYVLITGFMKGGVFQGGNYALKIDRTPLNSNGVDIDDAGRSPNNASTNDSHEGAGFDDTPADAKSKGNAWGQEMGCNLVAHPIGTPPANVDLDWFYVEYPY
GLTDGNYLDDPEANNADYGLSITDALRAYKEIGYGDKTVVAGIIDTGINMKHKDFKDENGESIVLYAKSCATDGTGGTYIGDGNPFTEIPIGLNWDKGAHGTHCSGTIAARGNNNIGIAGVAWKNTKLISYQSLGVEGSGRTWSVYGAMADLTKIVKILRKPKANRSVAENNALPSYLQNTDFQITQETVPVNMSLGGSYGSEFAFAVLTNAVKHNILPVIAMGNEGRYTAAYPAAFPGVLAVGATNGKDKKVHFSNSGAWISVSAPGDGIKSCGIHGEDDYETMSGTSMATPFVTGTIAYLLSFDNAHKITPYQMKALLEKTADKVDGATDFTDSLGHGRVNVYKAAKAIKDGNIPAPNDIYTEAEVTVTVKNNDGHSNDIVPCKITLVDEETHAPLAYVAGTGSNPPAFKGLIKGRSYSVYGAFLGSSSNQTFTAQDVDNPITIQFNKKIVWVSTVPNLHYNSGDDDTDTRIMVYKADASGNLDLNTAQSIVDYDQDLLDTTCFEAETGAKYYVLITGFMKGGVFQGGNYALKIDRTPLNSNGVDIDDAGRSPNNASTNDSHEGAGFDDTPADAKSKGNAWGQEMGCNLVAHPIGTPPANVDLDWFYVEYPY
Dentilisin/PrtP
Dentilisin/PrtP
Nisin z
N-terminal domain
C-terminal domain
Hinge
Hinge
C-terminal domain
N-terminal domain
Nisin z
ITSISLCTPGCKTGALMGCNMKTATCNCSIHVSK
ITSISLCTPGCKTGALMGCNMKTATCNCSIHVSK
Supplementary Fig. 6. Predictive docking models of nisin with dentilisin/PrtP and pro-MMP2. Panels (a) and (b): predicted hydrogen bond interactions between mature PrtP (N-terminal domain aa1-aa158 removed) and nisin including Knisin aa34-Ddentilisin aa240 (confidence score: 0.79). Panels (c) and (d): predicted nisin-MMP2 docking interaction showing no hydrogen bond contacts at the interface between PrtP and pro-MMP2 shown in Supplemental Fig. 4 (confidence score: 0.71). Panels (e) and (f): Predicted interaction among PrtP, pro-MMP2 and nisin. Panels (a), (c) and (e) are 3D structure videos. Panels (b), (d) and (f) are static docking models. For reference purposes, amino acid sequences of mature PrtP and nisin are included in Panels (b) and (f).
