# Supplementary material for: Nisin bacteriocin blocks T. denticola-triggered MMP2 activation and pathogen internalization via TLR2
Source: Sci Rep. 2026 Mar 11;16:13085. doi: 10.1038/s41598-026-43673-8 (PMC13100033; doi:10.1038/s41598-026-43673-8)
Supplement: Supplementary file 2 — Supplementary Material 2 [file 41598_2026_43673_MOESM2_ESM.pdf]

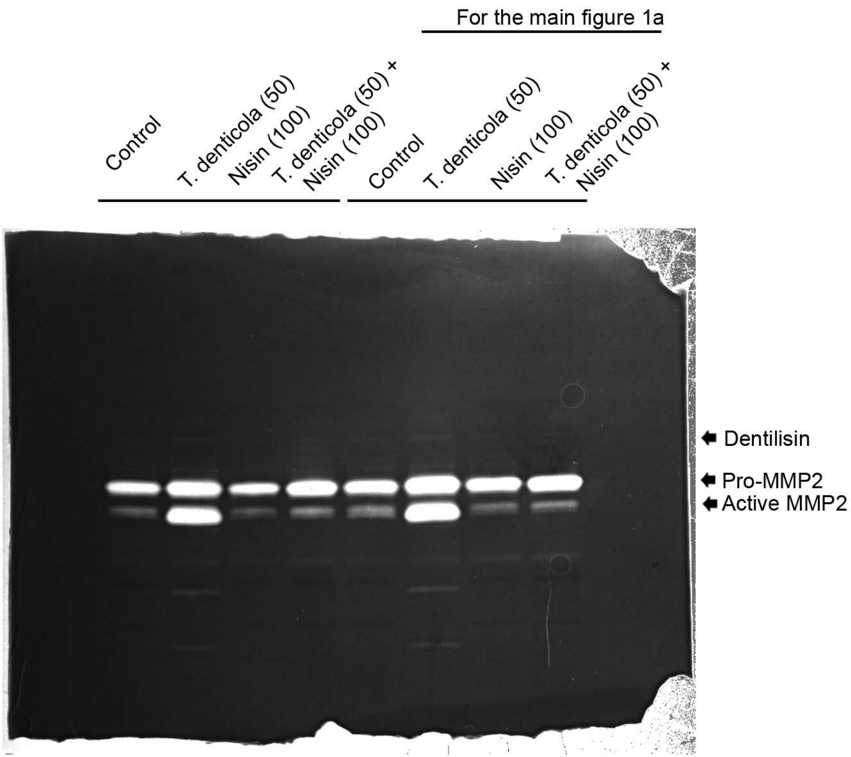

Figure 1a. Nisin inhibits *T. denticola*-induced-MMP2 activation in PDL cells. Representative gelatin zymogram showing MMP2 levels in PDL cells exposed to wild-type *T. denticola* (35405) (50 MOI) for 2 h, washed three times with PBS, followed by treatment with nisin (100 μg/mL) for 24 h. Conditioned media were analyzed by gelatin zymography.

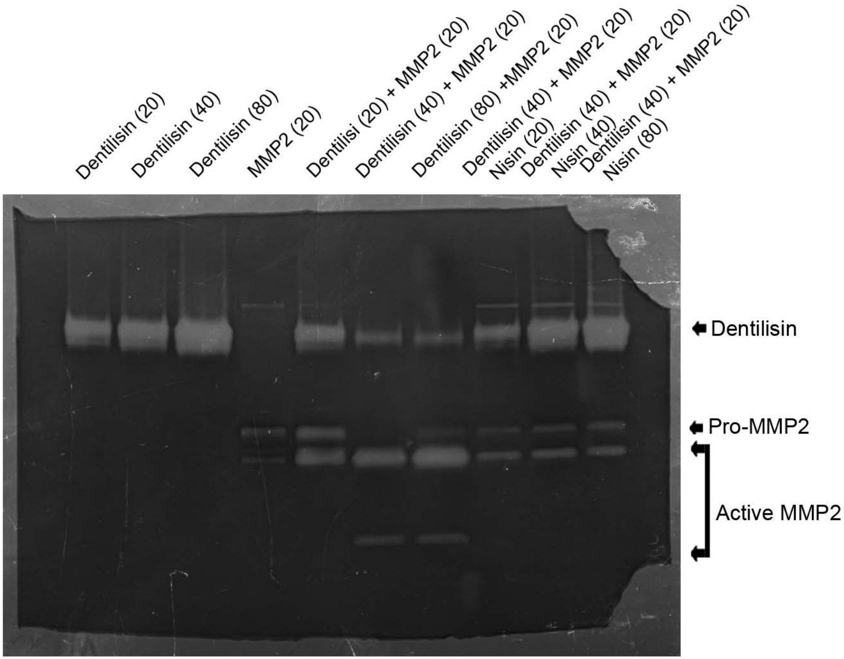

Figure 2a. *T. denticola* purified dentilisin directly activates recombinant MMP2 *in vitro*, and nisin attenuates this activation. Representative gelatin zymogram of recombinant Pro-MMP2 (20 ng) incubated with increasing concentrations of purified dentilisin (20, 40, or 80 ng) in the presence or absence of nisin (20, 40, or 80 μg).

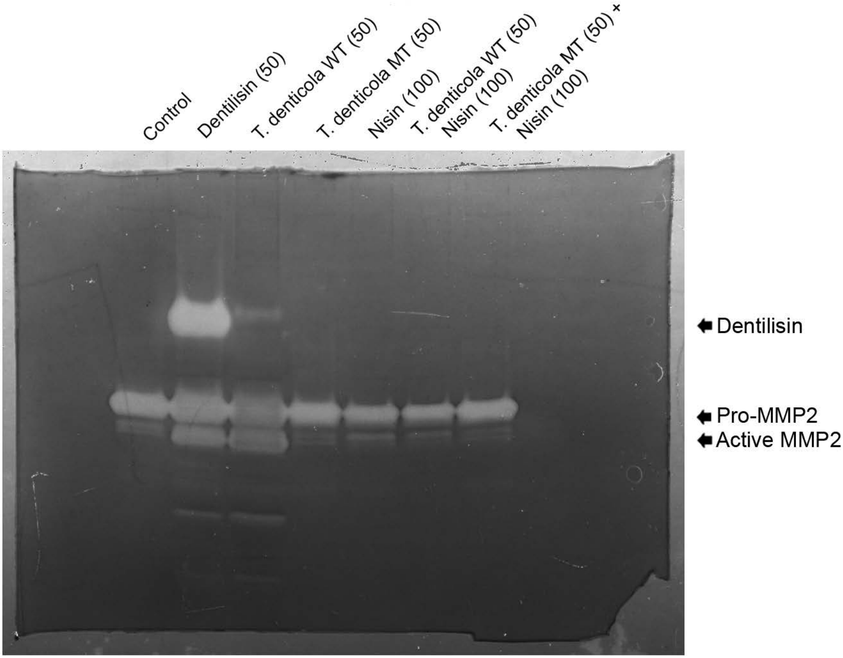

Figure 3a. Dentilisin-deficient *T. denticola* does not activate MMP2 and is unaffected by nisin in PDL cells. Representative gelatin zymogram showing MMP2 levels in PDL cells exposed to purified dentilisin (50 ng/ml), wild-type *T. denticola* (WT) (35405) (50 MOI) or mutant *T. denticola* (MT) (50 MOI) for 2 h and treated with nisin (100 μg/ml) for 24 h.

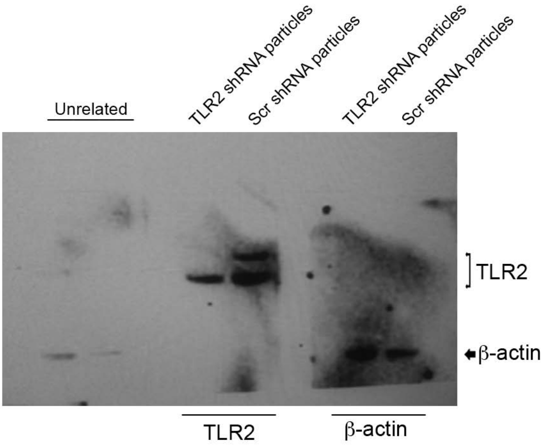

Figure 4a. PDL cells were transduced with TLR2 shRNA or scramble shRNA lentiviral particles in serum-free media, then selected in puromycin. (a) Immunoblot showing TLR2 and β-actin protein levels in cells transduced with TLR2 shRNA or scramble shRNA lentiviral particles.

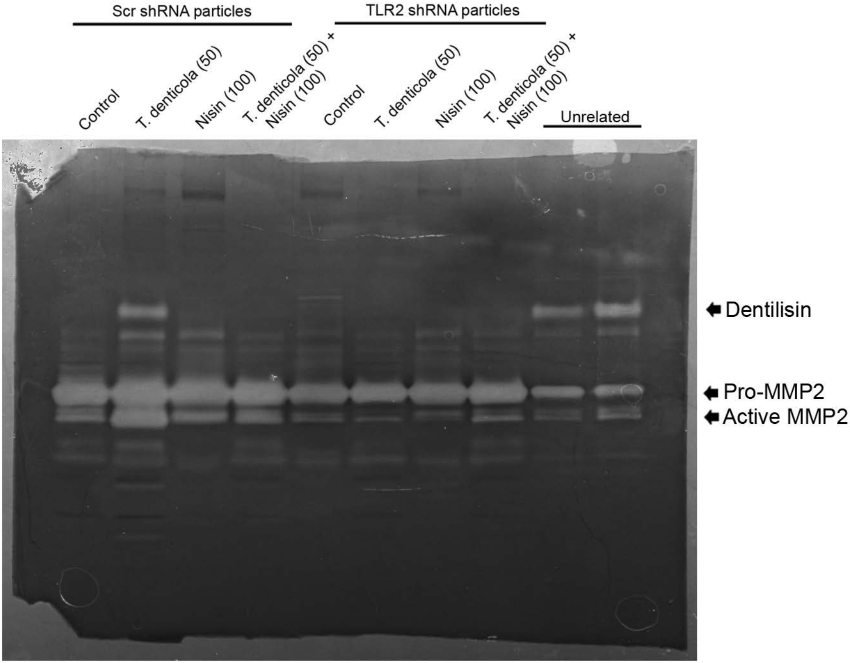

Figure 4b. *T. denticola*-triggered activation of MMP2 and nisin effects are mediated via TLR2 in PDL cells. Representative gelatin zymogram showing MMP2 levels in PDL cells exposed to wild-type *T. denticola* (35405) (50 MOI) for 2 h, washed three times with PBS, treated with gentamicin (50 μg/ml) for 1 h, washed three times again with PBS, and then treated with nisin (100 μg/ml) for 24 h. Conditioned media were analyzed by gelatin zymography.

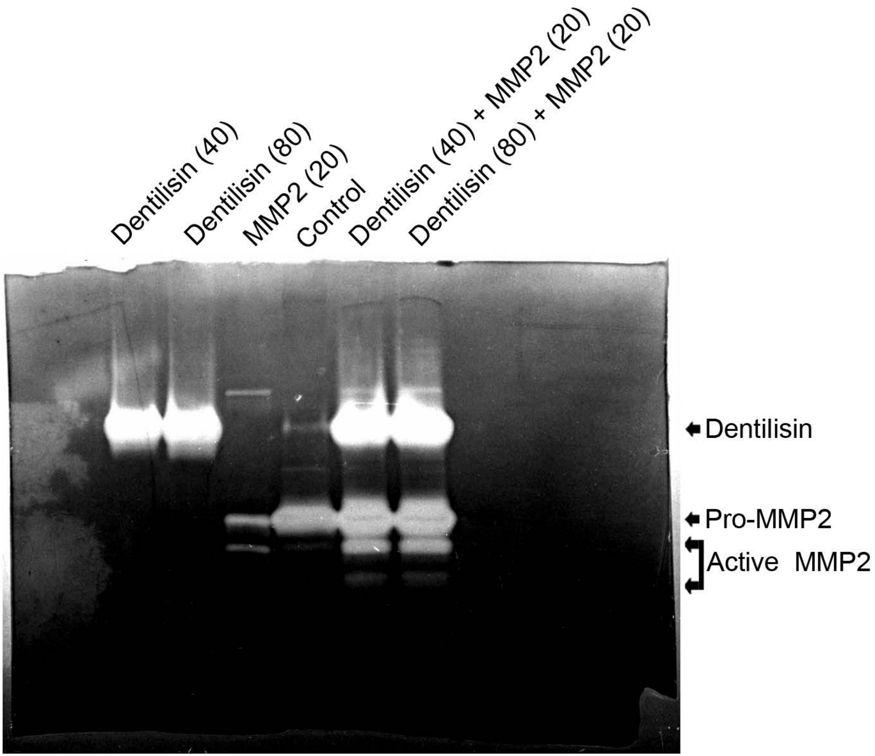

Supplementary Figure 1a. Dentilisin gelatin-degrading activity persists in the presence of PDL cells. (a) Gelatin zymogram of purified dentilisin (40 or 80 ng), recombinant Pro-MM2 (20 ng), or conditioned media from PDL cells treated with dentilisin (40 0r 80 ng/ml) and recombinant Pro-MMP2 (20 ng/ml) for 24 h.
